# Barriers to implementation of a computerized decision support system for depression: an observational report on lessons learned in "real world" clinical settings

**DOI:** 10.1186/1472-6947-9-6

**Published:** 2009-01-21

**Authors:** Madhukar H Trivedi, Ella J Daly, Janet K Kern, Bruce D Grannemann, Prabha Sunderajan, Cynthia A Claassen

**Affiliations:** 1 Mood Disorders Research Program & Clinic, Department of Psychiatry, University of Texas Southwestern Medical Center at Dallas, TX, USA

## Abstract

**Background:**

Despite wide promotion, clinical practice guidelines have had limited effect in changing physician behavior. Effective implementation strategies to date have included: multifaceted interventions involving audit and feedback, local consensus processes, marketing; reminder systems, either manual or computerized; and interactive educational meetings. In addition, there is now growing evidence that contextual factors affecting implementation must be addressed such as organizational support (leadership procedures and resources) for the change and strategies to implement and maintain new systems.

**Methods:**

To examine the feasibility and effectiveness of implementation of a computerized decision support system for depression (CDSS-D) in routine public mental health care in Texas, fifteen study clinicians (thirteen physicians and two advanced nurse practitioners) participated across five sites, accruing over 300 outpatient visits on 168 patients.

**Results:**

Issues regarding computer literacy and hardware/software requirements were identified as initial barriers. Clinicians also reported concerns about negative impact on workflow and the potential need for duplication during the transition from paper to electronic systems of medical record keeping.

**Conclusion:**

The following narrative report based on observations obtained during the initial testing and use of a CDSS-D in clinical settings further emphasizes the importance of taking into account organizational factors when planning implementation of evidence-based guidelines or decision support within a system.

## Background

### Difficulties associated with implementing evidence-based care

Although state of the art evidence-based clinical guidelines have been available to clinicians for some time, it is now well understood that the implementation or adoption of research information in actual clinical practices lags far behind, with limited potential to change physician behavior [1-3]. Unfortunately to date, efforts to implement quality improvement (QI) research findings into practice have often proceeded without insistence on the same level of rigor required to establish these QI targets as worthy of implementation [4]. The capacity to base treatment decisions on state-of-the-art knowledge alone appears to be insufficient to motivate behavior change among clinicians. Despite this, passive dissemination of evidence-based approaches in the form of instructional workshops or written manuals is the most frequently used approach to implementation of evidence-based guidelines in real-world settings [5].

While there is evidence that studies have demonstrated improved outcomes when guidelines are introduced during a research project [6], unfortunately benefits dissipate after the research program is over [7]. While Katon et al. found improved outcomes for major depressive disorder (MDD) in a primary care setting when initially implemented [8], after the research-based support system was removed, primary care physicians returned to pre-study levels of guideline adherence with respect to appropriate medications and dosage levels [9], with clinical outcomes no longer significantly improved compared to pre-intervention.

### Existing solutions

In terms of potential solutions, a number of strategies have been suggested to encourage the use of clinical practice guidelines [5], including multifaceted interventions involving audit and feedback of treatment practices, reminders about appropriate use of guidelines, local consensus processes in adoption of guidelines, as well as interactive educational meetings. It has been suggested that while specific strategies will likely be most effective in generating practice change, the methods to maintain change must also be in place, with system- and organization-level factors being critical in sustaining change [10]. Solberg [11] notes that previous reviews on this topic have neglected to address contextual factors that could affect implementation such as organizational support (leadership procedures and resources) for the change, and agency-level strategies needed to implement and maintain new systems.

It is now recommended that implementation of guidelines or other practices should not be undertaken without addressing these contextual factors [12]. Yet, despite the importance of such organization-level contextual factors, the impact of these implementation factors has generally been neglected [13,14].

### Barriers to implementation

A recent systematic review of the literature identified barriers to guideline adherence, including a lack of: awareness, agreement, or perceived self-efficacy to change; minimal outcome expectancy; and an inertia associated with faith in existing treatment practices [13]. In addition, external barriers such as lack of time, insufficient staff/support, and patient-related factors were also listed. The authors concluded that it is important to be aware of these barriers so that they may be overcome. One essential factor in successful implementation is physicians' faith in the algorithm itself, particularly in light of the common physician concern about losing autonomy in treatment decision-making [15]. Acceptable, built-in, algorithm-based treatment alternatives – rather than a single treatment mandate – increase adherence by permitting physicians some level of treatment choice [14]. Algorithms must also be easy to use and understand, as well as time efficient, in order to be sustainable. Margolis et al. showed that although physicians acknowledged making suboptimal decisions prior to algorithm implementation, they found adhering to it to be too tedious [14].

### Computerized Decision Support Systems (CDSS) – a potential solution

By using novel health information technology (IT), researchers can bring guideline information to the exact point within the clinical treatment process where decisions are being made [16,17]. Recommendations have previously been made for accelerating the development and adoption of a computerized decision support system (CDSS) for evidence-based medicine [18], with the advantage of potentially providing a strategy for guideline implementation that will be more sustainable for clinical practice (Additional file 1).

The majority of published studies demonstrate that health information technology components positively impact chronic illness care [19]. Aspects of health IT systems found to be correlated with enhanced health outcome included linkage between the technology system and an electronic medical record, computerized prompts during treatment decision-making, availability of progress reports and feedback, specialized decision support, electronic patient appointment scheduling, and access through the technology to patient health records. In contrast, barriers to the use of these technologies included cost, data privacy and security concerns, and inability within the technological system to adapt to rapid clinical workflow. A recent article reports on a survey of factors affecting clinician acceptance of clinical decision support – revealing that even though a majority of the clinicians were not explicitly following clinical support suggestions provided, they did feel that such systems are of benefit and reported that they would be even more beneficial if they had more time to make use of them [20].

Our own group has developed a CDSS based on the Texas Medication Algorithm Project (TMAP) treatment algorithms developed for MDD [21], schizophrenia [22] and bipolar disorder [23]. Our computer platform lends itself to instant consultation during the care of patients with any of these disorders. The following narrative report describes the barriers encountered during the original pilot testing conducted in the public health sector following development of the CDSS program for MDD (hereafter known as CDSS-D). Implementation provided an opportunity to assess the feasibility of using CDSS-D in a busy public mental health setting with a wide range of patients. It also provided an opportunity to integrate the software with a variety of administrative and billing programs and to receive feedback from "real world" clinicians on the utility of the program.

### Aims

1) To assess the feasibility of implementing a CDSS in real world practice;

2) To identify barriers to its implementation; and

3) To evaluate its impact on workflow issues in busy clinics.

## Methods

### Development of the CDSS-D

The CDSS-D was developed at the University of Texas Southwestern (UTSW) Medical Center, Dallas. The design of the CDSS-D is such that computer interaction is intended to be efficient and advantageous to the clinician by establishing clinical decision making for treatment through the computer as a by-product of every day clinical practice. The decision support system was developed utilizing the guidelines derived from those of the Texas Medication Algorithm Project (TMAP) [21-23].

### Description of the CDSS-D platform

The CDSS software program can be loaded on any personal computer with the recommended system requirements. The CDSS consists of three separate parts responsible for user interaction, decision-tree reasoning, and storage of clinical data. The relationships between these three parts are as follows:

***The user interface ***– is an interactive application written for Microsoft Windows and developed using Visual Basic programming language. Users can navigate through Web-like buttons that provide a user-friendly environment in which to work. The user interface is the only application of the program that is visible to the user.

***The rules engine ***– is derived from the clinical algorithms developed by TMAP, which have been translated into specific "rules" by its developers and UTSW computer information systems (CIS) personnel. They have been compiled into a knowledge base and implemented using industry-standard logical inference engine licensed from FairIsaac Software. The Rules Engine application operates behind the user interface to apply the TMAP algorithms to current and historical patient data to provide treatment options to the physician via the user interface.

***The CDSS database ***– contains clinical information entered into the CDSS application and is securely stored in the back-end SQL server. The database also stores user-specific data (for limiting access to clinical information) and the reference tables for medications and doses. Because both the reference tables and the rules knowledge base by which the rules engine processes the patient information are stored on a central server, updates to the algorithms can be implemented through the server, without user intervention. The CDSS provides assistance in diagnosis and decision support with appropriate treatment choices, follow-up, and preventive care, while at the same time providing access to physician order entry, alert systems, electronic documentation, and information retrieval.

### Information technology (IT) requirements for the CDSS

The computer software program has minimal requirements. The CDSS program requires Win2000/WinXP/Win2003 operating systems, Microsoft Internet Explorer 5.5 or higher, and TCP/IP connectivity to the server. In addition, the program requires that each personal computer (PC) be current with Microsoft Windows updates. Other minimum requirements may be applicable depending on how the program is being implemented within a specific environment. For example, if the software and database are loaded on one central server and end users access it through a Web-based portal, minimum levels of speed, bandwidth, and system memory can affect performance, as they would with any software application that transfers data bi-directionally via a Web connection.

### Sites and participants

To examine the feasibility of implementation of CDSS-D and its effects in routine clinical care, five public mental health clinics in Texas participated as clinical sites. Participating sites were recruited after the senior author (MHT) had made contact with local senior medical directors as part of the process of rolling out the Texas Medication Algorithm Project (TMAP). A group of these senior medical directors subsequently expressed interest in being involved with field-testing of CDSS-D. All five sites participating were representative of urban public mental health care in Texas. Fifteen study clinicians (thirteen physicians and two advanced nurse practitioners – APNs) participated across five sites. Participating clinicians were approached by their local medical director and invited to participate if they were interested. The majority of the clinicians approached chose to participate. The field-testing consisted of over 300 outpatient visits by 168 patients, all of whom had clinician-diagnosed MDD based on the Diagnostic and Statistical Manual-Fourth Edition-Text Revision (DSM-IV-TR) [24].

### Site setup and information technology support

Prior to implementation, the medical directors at each site approached their own IT department to request support for the CDSS-D program. At that point the local IT department was given the program requirements by the UTSW developers. Site set-up for the CDSS-D program started one month before implementation at the clinic. Clinical Information Systems programmers and software developers from UTSW were responsible for data management on the central server and troubleshooting for local hardware and software system installation and support. At some sites, computers and programs had to be upgraded. Instruction on the use of the software was provided during initial physician training, and trained research assistants were available on-site to provide technical assistance to the clinicians with the initial implementation of CDSS-D during normal clinic operating hours. In addition, both technical and medical help desks were available via telephone to software users on a 12 hour/day, 5 day/week basis.

The CDSS-D can be deployed as a single-user environment whereby a single personal computer (PC) can run all the components – client and server. In this case, testing was generally limited to ensuring program compatibility with the individual PC and successful installation of the software in a working condition. The CDSS-D program was also designed to support multiple local/remote clients. In this case, a much more comprehensive integration and testing process was required. In order to ensure successful integration, a test database or a "test environment" was set up and the real life use of the program was simulated. In this approach, testing of the inbound and/or outbound parts of the interface was also regarded as essential.

### Training of clinicians

Prior to receiving training on the use of the CDSS-D program, clinicians first received extensive training regarding the guideline for treating depression. As part of this process, the senior author (MHT) gave several lectures reviewing treatment strategies for MDD and highlighting the benefits of algorithm-based care. Initial training, conducted by the project's training director (JKK), included a thorough review of treatment strategies for the remission of depressive symptoms, education about the TMAP algorithm for MDD, and hands-on practice in the use of the computerized algorithm. Simulated visits were created to illustrate how the algorithms are used in daily practice. The educational aim of the training workshops was to help participants understand the algorithm, the algorithm stages, and the critical decision points. Additional instruction in the use of CDSS-D software lasted about 4 hours, and involved both demonstration and hands-on training. To ensure a basic knowledge of how to complete a patient visit, each clinician was asked to enter an initial and follow-up visit using simulated patient material in a step-by-step fashion with the instructor, followed by independently entering several additional case scenarios.

At the end of the workshop, each clinician was given a written instruction manual, which could also be accessed on the user interface screen when using the program. Clinicians were also instructed in how to access and use the technical support. In addition, research associates familiar with CDSS-D were available on site initially and later by phone to provide clinicians with real-time support as needed.

### Support staff training

Anticipating workflow transition issues, we also held pre-implementation training sessions for program directors and clinic managers so that they would understand the program and be able to suggest solutions to potential issues and approve any fundamental change in the clinic process that might be required.

### Feedback from clinicians

During the period of field-testing, a series of informal qualitative interviews took place between the training director for the project (JKK) and the participating clinicians and support staff at the sites. It should be noted that during this time the CDSS-D program underwent various modifications so that the participating clinicians worked on multiple versions or builds. After each new build was rolled out and field-tested, the training director spoke to the participating clinicians to obtain their feedback and any necessary additional modifications were made at that point. The following section provides an account of the issues the clinicians and support staff reported.

## Results

The following describes the experience, including constraints and problems encountered, and feedback received at the five participating public health clinical sites.

### Barriers to implementation of the CDSS-D

#### Computer Literacy

During this pilot testing we encountered wide variation in levels of computer literacy and confidence among clinicians at the five participating sites, as well as in typing skills. In general, clinicians who were accustomed to using computers in their daily practice adapted better.

For many clinicians, technical errors encountered during the introduction and early use of the software program frequently precipitated a loss of confidence with the program. While some clinicians appeared confident in dealing with software error messages, others were not willing to tolerate the experience during a clinical visit. Additionally, clinicians reported increased frustration in those clinics where they had to move between two software systems for each patient visit (i.e., the CDSS-D system and the clinic's own electronic medical record).

#### Technical Requirements and Availability of On-site IT Support

We found that technical support to maintain the server and network was crucial to smooth operation and interfacing speed. If the server or network was inadequate, the program became too slow for clinicians to use during busy patient contact times. "Screen loads" – the time that it takes for the next screen to become completely visible and usable to the user – varied by implementation site and from clinic to clinic. For example, at one site, when the CDSS-D program was loaded onto a shared server, the program slowed to the point of becoming unusable. At another site, though the CDSS-D had its own server, the network had inadequate bandwidth, and the screens loaded six to ten times slower at the branch clinics than at the central site. At sites where the program was configured to interface with other client record systems, successful implementation depended on the local IT staff commitment to work with UTSW computer support staff to create and maintain a bridge between the two software systems.

#### Site-specific Issues

Clinic processes and clinic requirements for treatment providers and support staff varied within each system and from clinic to clinic. Established workflow systems often centered on the structure and format of required clinic paperwork. Adaptations had to be made for routine tasks such as requests, approval, and documentation of medication refills and changes outside of scheduled doctor visits. At times, clinicians and support staff voiced frustration when moving from paper and pencil to electronic record. As a result, at times, system changes were necessary which had to be approved by administration.

Similarly, different sites used different billing codes and billing software, and the required form names and numbers for the CDSS-D electronically recorded progress notes varied. Certain requirements were met immediately by making changes to the configuration files; however, ultimately, we had to add a "customization tab" within the program so that sites could configure other files to meet these site-specific requirements.

#### Buy-in and Support from Administration

In general, while there was full support and buy-in from the local senior medical directors, this was not always the case at the local administrator level, and likely was another factor impeding the implementation process.

#### Clinician Autonomy and Flexibility of the Decision Support System

In order for the software program to be used in all cases in "real world" settings, we found that considerable treatment flexibility was necessary. For example, public health settings frequently include large numbers of difficult-to-stabilize patients who had been in treatment for a long time on an older medication not included in the state-of-the-art TMAP algorithm because of poor efficacy or tolerability issues. Understandably, in certain cases clinicians did not want to change the medication to an algorithm medication when the patient was stable and without problems. In addition, sites often had specific medication formularies that included some of the algorithm medications and excluded others. There were also formulary issues based on payment sources, such as Medicaid and private insurance companies.

#### Impact on Patient Attitudes

Despite prior concerns that computer use may depersonalize the clinical encounter, decrease eye contact, and be distracting, feedback from patients in the form of surveys revealed that patients in general felt comfortable with the care being provided by physicians using the CDSS-D. A separate manuscript describing the findings from the patient surveys is currently in progress.

### Impact on workflow

Early involvement from all key personnel whose departments would be affected by a transition from pen and paper documentation to an electronic medical record system was essential for successful implementation. Making the transition at a single point in time from pen and paper to an electronic system was the preferred approach, rather than trying to change gradually by operating two systems simultaneously, with clinicians reporting both frustration and confusion when asked to use both systems.

One of the primary constraints encountered in the public mental health sector was the fact that the average time allocated per patient visit was only 15 minutes. As with the use of any new software in a work environment, the start-up period was associated with an initial drop in productivity, making it difficult for clinicians to see the same number of patients per day using the regular clinic schedule. Where clinician pay schedules are attached to "productivity" (i.e., the number of patients seen per workday), these issues become substantial.

In an effort to overcome this barrier, we tried to unfold the program gradually into the practice by having clinicians use CDSS-D with only one or two patients per day while in training, though it is possible that this approach impacted the clinicians' ability to become familiar or comfortable with the program quickly.

## Discussion

Despite wide promotion, clinical practice guidelines have had limited effect in changing physician behavior [1-3]. Previous guideline research indicates improved outcomes when guidelines are adhered to during a research project [6], but benefits dissipate after the research program is over [7].

The issues raised by clinicians and support staff are similar to those reported previously, including concerns about lack of time and the impact of the program on clinical workflow. Issues that impacted the use of the program can be placed into hierarchical categories: (1) issues that prevented the use of the program, (2) issues that discouraged the use of the program, and (3) issues that make the program more convenient to use.

In regard to issues that prevented the use of the program, generally these issues were due to process or workflow conflicts. For example, earlier programs made all of the medications in the Texas Medication Algorithm Project (TMAP) available for use; however, some patients arrived to be treated who were stable and doing well on older psychotropic medications that were not in the algorithm. Clinicians were at a loss as to how to enter the patient into the program. In addition, earlier programs lacked an effective way to titrate, taper, and postpone the start of a medication. These issues prevented the use of the program and were all corrected with subsequent modifications.

In regard to issues that discouraged the use of the program, though these issues generally made using the program difficult, they did not stop the clinician from using the program. For example, if clinicians changed their minds regarding the choice of medication during the visit and returned to the medication page to make the change, all of the notes on subsequent pages would erase and the clinicians would have to rewrite the notes. Program speed and reliability also fell into this category. Issues like these were frustrating for clinicians and again were all corrected with subsequent modifications.

In regard to issues that made the program more convenient to use, generally these consisted of minor program extras. For example, clinicians wanted spell-check, links to an electronic Physician's Desk Reference, and more editing ability in all fields.

Other barriers identified related to specific-site issues and the availability of adequate IT support. Despite the barriers encountered, in terms of feasibility of implementing the CDSS-D, during this initial testing period physician use and compliance with the CDSS-D increased after initial hesitation with the addition of components of IT integration, training, and on-site support. In addition, a number of barriers to change were eliminated through continued modification of the CDSS-D. Increased training time and IT support also aided in the successful implementation. Overall, clinicians reported finding the CDSS to be a useful tool in providing good clinical care. See Tables 1 and 2

**Table 1:** Lessons learned during implementation of a computerized decision support system

Lesson Learned	Solution Proposed
Variation in computer literacy may affect adherence.	Enhanced training on the computer program, so that clinicians achieve necessary competence and have used program on multiple simulated cases.

Adequate IT requirements to support the program need to be in place.	Prior to and following implementation, appropriate IT support to maintain server/network is crucial.

Availability of on-site technical support needed.	Training of individuals at site who can provide extended support for clinicians and support staff as needed.

Management and administrative support and involvement essential to assist with impact on workflow.	Include management and administrative staff in planning to assist with issues such as visit frequency and other potential workflow issues.

Software may need to be adapted to specific site workflow/administrative issues.	Prior to implementation, work with site personnel to evaluate specific site requirements and allow customization.

The need to allow clinicians flexibility and autonomy in the use of the algorithm.	The algorithm provides clinicians with recommendations that they can choose to over-ride.

Clinician feedback essential to making the program more usable.	Prior to implementation, a period of testing allows clinician feedback and any necessary program modification.

A summary of lessons learned during initial testing of the Computerized Decision Support System for Depression (CDSS-D) in real world clinical settings

**Table 2:** Essential requirements for implementation of a computerized decision support system

User-friendly
Provides decision support at the point of care

Allows clinician flexibility in treatment decision-making

Central server facilitates program updates

Preferably seamless connection to an Electronic Health Record

Easy access to IT support

Adequate initial hands-on training and easy-to-use written documentation and reference materials

Host hardware and software system that allows adequate processing speed for clinical workflow

Readily modifiable reports to meet local site specifications

A summary of the essential requirements for successful implementation of a Computerized Decision Support System for Depression (CDSS-D) in real world practice settings

Examples of some of the modifications made in order to increase the likelihood of obtaining support on all levels within the clinic system include the following: addition of a demographic screen to facilitate search by patient number; addition of diagnostic codes for billing purposes; modification of the prescription screen to allow titration, tapering and splitting; and addition of a facility to customize billing on the finishing screen. All of these modifications came about as a direct result of specific requests from the sites and were implemented to facilitate the integration of the CDSS with current site-specific administrative and clinical procedures.

A recent editorial suggests that health information technologies are tools whose value is influenced by how clinicians modify their work practices to use them and how organizational change is enacted when they are adopted [25]. Even though organizational factors were not examined in depth in this report, the investigators who conducted and monitored the field-testing did report difficulties that likely related to the fact that there may not have been full buy-in from administrators at the sites themselves. The importance of this factor in the implementation process has become increasingly recognized since our early field-testing.

Other limitations of the current study include the small sample size and the fact that the information received was in the form of informal feedback from participating clinicians rather than surveys or questionnaires. Specifically, the small sample size and the fact that all participating sites were urban means that the feedback received may not be generalizable to other public mental health sites.

## Conclusion

Based on reviews to date, there is now increasing evidence that for a change to work and be maintained the relevant clinicians must have the knowledge, skills, and motivation to implement a practice; practical and organizational factors must make the practice feasible; and colleagues, patients, and other important stakeholders must accept it.

Many of our observations during the initial testing and use of CDSS-D in clinical care further emphasize the importance of taking into account these factors when planning for the implementation of computerized, evidence-based guidelines and decision support within a system of care. From our own experience, for clinician behavior to change, the associated changes within the system need to be anticipated and planned for carefully on a site-by-site basis with full support and buy-in from administrators from the outset.

Based on our experience with field-testing CDSS-D, we have incorporated some of the lessons learned when initially planning the implementation of more recent versions of CDSS-D in real world practice settings. Additional file 2 shows an example of clinician surveys related to the usefulness of the CDSS that were used during the original field testing. In conjunction with individual comments from participating clinicians, feedback received from these surveys helped developers with subsequent modifications.

Other recent strategies developed to facilitate this process have included detailed needs assessment surveys and focus groups conducted with stakeholders, administrators, clinicians, and support staff, with a goal of identifying potential barriers and obtaining support for the project at all levels prior to implementation.

We also strongly agree with a recent editorial suggesting that while adoption of technologies is often assumed to be a single event marked by a distinct before and after, it is in fact a multistage process that involves the routinization of the technology after it is implemented [25]. With this in mind, in our most recent project with the most recent version of CDSS-D, the clinical support system was merged with an existing electronic health record in a public mental health care system so that, if successful, it will remain in place well after the research has been completed and become a routine part of the system of care.

### Future direction

Of note, the American College of Medical Informatics (AMIA) in June 2006 released a roadmap for national action on clinical decision support [26]. The AMIA report addresses the limited extent to which clinical decision support is being used to improve healthcare, identifying six strategic objectives as a potential means of achieving widespread dissemination and use of effective clinical decision support within the U.S. healthcare system. Among these objectives: representing clinical knowledge and clinical decision support interventions in standardized formats (both human and machine-interpretable); addressing policy/legal/financial barriers and creating additional support and enablers; and finally, advancing care-guiding knowledge by fully leveraging the data available in interoperable electronic health records (EHRs) to enhance clinical knowledge and improve health management [26].

Our experience with the issue of adherence to paper-and-pencil algorithms in both TMAP and STAR*D, as well as data supporting the importance of providing clinician feedback at the time of the patient visit, further illustrates the potential benefits of a state-of-the-art computerized decision support system developed by our group for sequenced treatment approaches. Unlike a paper-and-pencil format, such a computerized system has the advantage of being able to provide evidence-based recommendations regarding medication dosing *at the point of care*, based on the clinician's assessment of symptoms and side effects at that clinic visit, thereby directly improving adherence to treatment guidelines. With this in mind and following a comprehensive needs assessment based on lessons observed during early testing of our CDSS-D in our most recent project as mentioned above, we plan on merging CDSS-D with an EHR in a public mental health system.

## Competing interests

Madhukar H. Trivedi, M.D. has been a consultant for Abbott Laboratories, Inc.; Akzo (Organon Pharmaceuticals Inc.); AstraZeneca; Bayer; Bristol-Myers Squibb Company; Cephalon, Inc.; Cyberonics, Inc.; Eli Lilly & Company; Fabre-Kramer Pharmaceuticals, Inc. Forest Pharmaceuticals; GlaxoSmithKline; Janssen Pharmaceutica Products, LP; Johnson & Johnson PRD; Eli Lilly & Company; Meade Johnson; Neuronetics; Parke-Davis Pharmaceuticals, Inc.; Pfizer, Inc.; Pharmacia & Upjohn; Sepracor; Solvay Pharmaceuticals, Inc.; VantagePoint; and Wyeth-Ayerst Laboratories. He has served on speakers bureaus for Abdi Brahim; Akzo (Organon Pharmaceuticals Inc.); Bristol-Myers Squibb Company; Cephalon, Inc.; Cyberonics, Inc.; Forest Pharmaceuticals; GlaxoSmithKline; Janssen Pharmaceutica Products, LP; Eli Lilly & Company; Pharmacia & Upjohn; Solvay Pharmaceuticals, Inc.; and Wyeth-Ayerst Laboratories. He has also received grant support from Bristol-Myers Squibb Company; Cephalon, Inc.; Corcept Therapeutics, Inc.; Cyberonics, Inc.; Eli Lilly & Company; Forest Pharmaceuticals; GlaxoSmithKline; Janssen Pharmaceutica; Merck; National Institute of Mental Health; National Alliance for Research in Schizophrenia and Depression; Novartis; Pfizer Inc.; Pharmacia & Upjohn; Predix Pharmaceuticals; Solvay Pharmaceuticals, Inc.; and Wyeth-Ayerst Laboratories.

Ella Daly, M.B. MRC. Psych., Cindy Claassen, Ph.D, Prabha Sunderajan, M.D., Janet Kern, Ph.D. and Bruce Grannemann, M.A. report no competing interests or disclosures.

## Authors' contributions

MHT conceived the study, participated in its design and coordination and drafted the manuscript. EJD, JKK, and CAC helped to draft the manuscript. PS worked on testing and technical modifications of the CDSS-D. BDG participated in the design of the study.

## Pre-publication history

The pre-publication history for this paper can be accessed here:

http://www.biomedcentral.com/1472-6947/9/6/prepub

## Supplementary Material

Additional file 1 **Contemporary Clinical Trials article (2007).** This article describes the design and rationale for Project IMPACTS (Implementation of Algorithms using Computerized Treatment Systems) – an ongoing, multi-site, NIH-funded study, which aims to evaluate how best to facilitate integration of depression treatment algorithms into routine psychiatric care. The article provides a full description of the Computerized Decision Support System for Depression (CDSS-D). Click here for file

Additional file 2 **CDSS Surveys.** Algorithm Evaluation Questionnaire: Software Application Version. Part I: Ease of Use Click here for file
