# Supplementary material for: Barriers to implementation of a computerized decision support system for depression: an observational report on lessons learned in "real world" clinical settings
Source: BMC Med Inform Decis Mak. 2009 Jan 21;9:6. doi: 10.1186/1472-6947-9-6 (PMC2639574; doi:10.1186/1472-6947-9-6)
Supplement: Additional file 1 — Contemporary Clinical Trials article (2007). This article describes the design and rationale for Project IMPACTS (Implementation of Algorithms using Computerized Treatment Systems) – an ongoing, multi-site, NIH-funded study, which aims to evaluate how best to facilitate integration of depression treatment algorithms into routine psychiatric care. The article provides a full description of the Computerized Decision Support System for Depression (CDSS-D). [file 1472-6947-9-6-S1.pdf]

Available online at www.sciencedirect.com
ScienceDirect
Contemporary Clinical Trials 28 (2007) 192–212
Contemporary Clinical Trials
www.elsevier.com/locate/conclintrial

Assessing Physicians' Use of Treatment Algorithms: Project IMPACTS Study Design and Rationale

Madhukar H. Trivedi *, Cynthia A. Claassen, Bruce D. Grannemann, T. Michael Kashner, Thomas J. Carmody, Ella Daly, Janet K. Kern

Department of Psychiatry, The University of Texas Southwestern Medical Center, 5323 Harry Hines Blvd., Dallas, TX, USA

Received 21 July 2005; accepted 1 August 2006

Abstract

Effective treatments for major depressive disorder have been available for 35 years, yet inadequate pharmacotherapy continues to be widespread leading to suboptimal outcomes. Evidence-based medication algorithms have the potential to bring much-needed improvement in effectiveness of antidepressant treatment in "real-world" clinical settings. Project IMPACTS (Implementation of Algorithms using Computerized Treatment Systems) addresses the critical question of how best to facilitate integration of depression treatment algorithms into routine care. It tests an algorithm implemented through a computerized decision support system using a measurement-based care approach for depression against a paper-and-pencil version of the same algorithm and nonalgorithm-based, specialist-delivered usual care. This paper reviews issues related to the Project IMPACTS study rationale, design, and procedures. *Patient outcomes* include symptom severity, social and work function, and quality of life. The *economic impact of treatment* is assessed in terms of health care utilization and cost. Data collected on *physician behavior* include degree of adherence to guidelines and physician attitudes about the perceived utility, ease of use, and self-reported effect of the use of algorithms on workload. Novel features of the design include a two-tiered study enrollment procedure, which initially enrolls physicians as subjects, and then following recruitment of physicians, enrollment of subjects takes place based initially on an independent assessment by study staff to determine study eligibility. The study utilizes brief, easy-to-use symptom severity measures that facilitate physician decision making, and it employs a validated, phone-based, follow-up assessment protocol in order to minimize missing data, a problem common in public sector and longitudinal mental health studies. IMPACTS will assess the success of algorithm implementation and subsequent physician adherence using study-developed criteria and related statistical approaches. These new procedures and data points will also allow a more refined assessment of algorithm-driven treatment in the future.
© 2006 Elsevier Inc. All rights reserved.

Keywords: Depression; Measurement-based care (MBC); Treatment algorithms; Physician decision support; Electronic medical records; Medical information technology

* Corresponding author. Mood Disorders Research Program & Clinic, The University of Texas Southwestern Medical Center, 5323 Harry Hines Boulevard, Dallas, Texas 75390-9119, USA. Tel.: +1 214 648 0188; fax: +1 214 648 0167.
E-mail address: Madhukar.Trivedi@UTSouthwestern.edu (M.H. Trivedi).

1551-7144/$ - see front matter © 2006 Elsevier Inc. All rights reserved.
doi:10.1016/j.cct.2006.08.005

1. Introduction and background

As is true of many disease processes, the real-world standard of care for the treatment of major depressive disorder (MDD) is often decades behind medical research. The Agency for Healthcare Research and Quality first published guidelines for the treatment of depression in primary care settings in 1993 [1,2], yet appropriate dosing strategies and duration of treatment remain the exception rather than the rule [3–7]. In reality, clinical care provided to patients with mood disorders varies significantly, with patients too often receiving suboptimal doses of antidepressant medications for insufficient periods of time [8].

Studies have consistently shown that the use of medication algorithms brings appropriate uniformity to treatments of MDD at predictable costs [9–12], thereby improving the quality and, likely, the overall cost of care. Despite their success, however, guidelines and algorithms have not been readily adopted in real-world care settings. They are often viewed as a threat to the physician's decisional autonomy [9,13–15] and as less than feasible due to the cost and time constraints associated with implementation [14]. Thus, while efforts to promote advances in technology and pharmacology have resulted in a surge of consensus guidelines and algorithms created for a wide variety of disease states [16,17], finding a dissemination method for these treatment approaches that would ensure their use in clinical settings continues to be a major challenge [13,14,18,19].

In a previous study (TMAP — Texas Medication Algorithm Project [8,20]) (see Fig. 1), we developed and tested a treatment algorithm for the pharmacological management of patients with major depressive disorder. To ensure that the algorithm was properly implemented in clinical care, an extensive physician support system was developed that included on-site clinical support personnel and expert consultation obtained via regularly scheduled teleconferencing. Compared to treatment as usual, treatment using the algorithm achieved better clinical outcomes but at higher costs, making the intervention impractical for many clinical settings.

Our previous experience with the TMAP study underscores the need to develop strategies to ensure efficacious, low cost implementation of algorithms. In addition, even in guideline-driven practice, both TMAP and its successor, the Sequenced Treatment Alternatives To Relieve Depression (STAR*D) trial [21,22], found that clinical treatment of depression varies widely, with clinicians often changing from one antidepressant to another too quickly or, conversely, conducting an unnecessarily prolonged treatment trial with an obviously unsuccessful medication or psychotherapy [20,23]. Practitioners also differed in how they assessed the outcomes of treatment (symptoms, function, side-effect frequency and burden), with global judgments often used instead of specific symptom assessments, even though the former are less accurate [24]. These differences led to wide variability in treatment implementation and likely also result in wide variations in outcomes in typical practice. To counter such problems, both the TMAP and STAR*D trials emphasized the importance of a measurement-based care (MBC) approach [23,25] wherein the physician assessed depression symptom severity, adherence to treatment, and potential side-effect burden at each visit and used this information when following the medication treatment protocol. We believe that an MBC approach is an essential component to any decision support system allowing the physician to individualize decisions about care for the patient based on their progress and their ability to tolerate the medication. MBC also allows the physician to use adaptive treatment strategies based on the individual patient's response to the chosen treatment approach.

One recently proposed approach to evidence-based MBC is to couple easy-to-use algorithms with computer-assisted decision support systems (CDSS) [26–31]. This approach makes state-of-the-art treatment information readily available to practicing physicians at the time of care, when it is most likely to improve the quality of treatment and, consequently, clinical outcomes [29]. We believe that the logical rules and specific data requirements inherent in most treatment algorithms can be effectively programmed into computer language, facilitating translation of state-of-the-art science into informed medical decision making at the time of service delivery [32,33]. Therefore, computerization of treatment algorithms is a viable solution to the obstacles confronting physicians when implementing algorithms in the public sector, and, as such, developing and testing such a system for delivery of treatment guidelines is a logical next step in facilitating the use of measurement-based, algorithm-driven, clinical practice in treating depressed patients.

Project IMPACTS compares patient outcomes and the cost of our previously developed paper-and-pencil-based pharmacological algorithm for the treatment of MDD with a computerized decision support system for depression (CDSS-D) based on the same algorithm. If found to be effective in the hands of psychiatrists, the system has the potential to be of immense use in primary care and family practice settings where the majority of depression care is delivered.

2. Methods

2.1. Study aims

Project IMPACTS is designed to examine the effectiveness of a computer-based decision support system for algorithm-based treatment of MDD (CDSS-D) compared to both a traditional paper-and-pencil-based, algorithm-driven system of care (PPA-D) and nonalgorithm-based expert usual care (UC-D). It will evaluate these three treatment methods across four domains: (1) patient outcomes, (2) direct cost of treatment, (3) impact on physician behavior and attitudes, and (4) overall health care cost versus outcome. Patient outcomes to be assessed include: symptom severity, social and work function, and quality of life. The economic impact of each approach to care is assessed in terms of overall service utilization cost (i.e., program costs for MDD-related care, other mental health care costs, and general medical care cost). Assessment of physician behavior and attitudes in both algorithm groups involves measuring adherence to treatment guidelines and perceptions about the usefulness, convenience, and effect on workload. By combining data from outcome and costs, we plan to conduct an overall cost outcome analysis at the end of the trial.

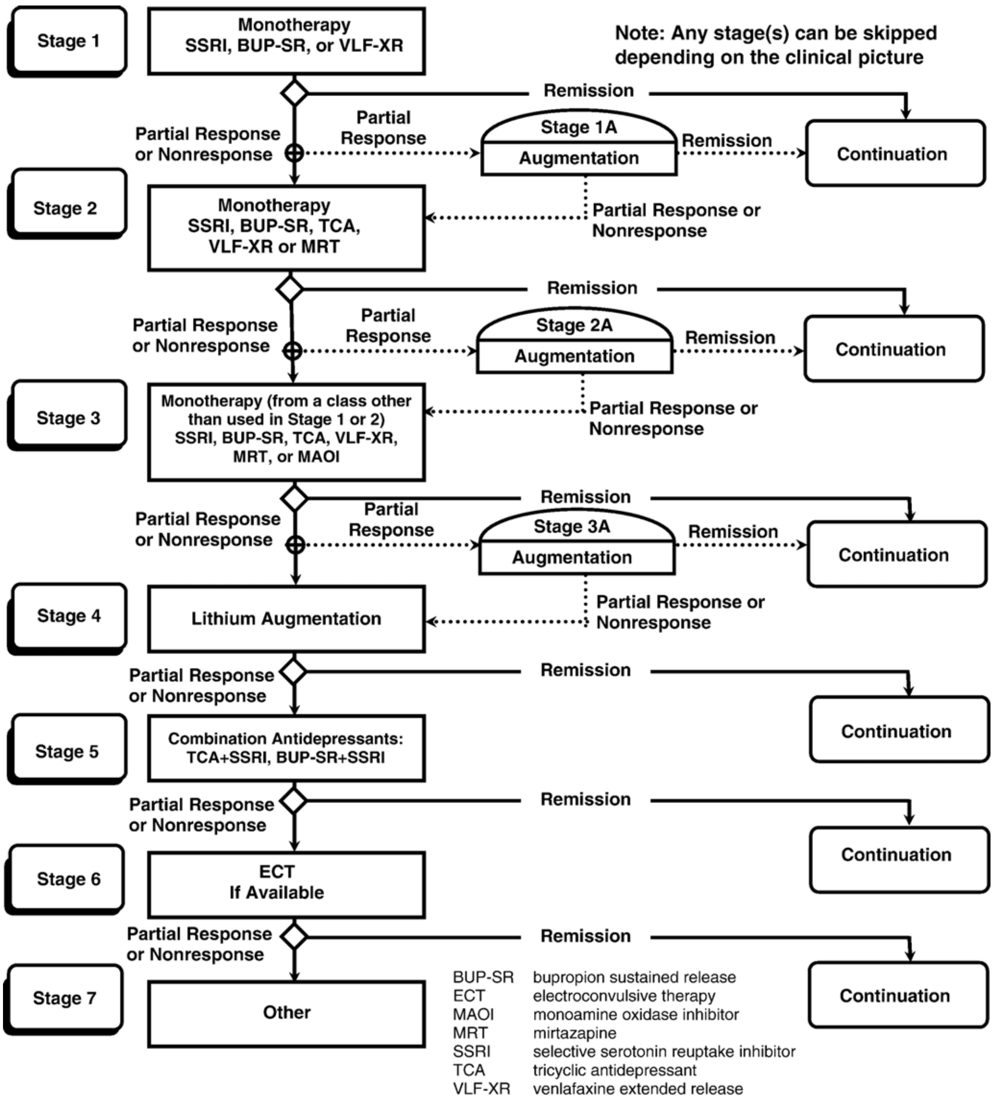

Fig. 1. Texas Medication Algorithm for the Treatment of Nonpsychotic Major Depressive Disorder.

2.2. Hypotheses

Although physician adherence to the algorithm is measured in this study, in the absence of improved patient outcomes, the integration of algorithm-based care into real-world treatment settings is meaningless. Primary study hypotheses, therefore, target patient outcome as opposed to measuring only algorithmic adherence under computerized and noncomputerized conditions. Specifically, we hypothesize that patients will have less symptom severity, better functioning, and higher quality of life when assigned to physicians using the CDSS-D than when treated by physicians using PPA-D. Similarly, better outcomes are predicted for patients assigned to the PPA-D physicians compared to the UC-D Physicians.

We anticipate higher costs for MDD-related care for patients in the CDSS-D group, compared to patients treated by PPA-D or UC-D physicians because of the increased visit frequency associated with algorithm-based care and increased adherence to the algorithm in the CDSS-D group. On the other hand, we hypothesize that better outcomes in the treatment of depression will lead patients to incur lower costs for other mental health and general medical care in the CDSS-D group, compared to PPA-D and UC-D-treated patients. Therefore, it is hypothesized that more favorable cost-outcome ratios will be achieved by patients treated by CDSS-D physicians compared to those treated by PPA-D or UC-D physicians.

2.3. Physician enrollment, randomization, and training

We will implement each of the three study arms in at least four different public health sector systems of care. The use of different public health care systems is designed to provide a greater degree of generalizability for our findings. A minimum of 15 physicians will be randomized to each of the three study arms. Each physician will treat 5–15 patients with MDD (total of at least 45 physicians and 540 MDD patients).

Because IMPACTS is also a study of physician behavior, physicians recruited to participate are likewise treated as human subjects. Physicians from each site who are interested in study participation have the goals, risks, and benefits of study participation explained to them. They provide written, informed consent and are afforded all related rights and protections, including voluntary participation, right to withdraw, and confidentiality of all data related to their study performance. The study's Data Safety Monitoring Board (DSMB) oversees both physician data and the traditional patient data for the safety and protection of all human subjects associated with the study.

Procedures for randomization of physicians to study arms take into account the need for cross coverage during physician absence in busy public sector clinics. This is accomplished using a yoked randomization scheme, which ensures that there will be at least one additional physician in the same condition on the same treating schedule to handle cross coverage for patient visits.

Prestudy training for enrolled physicians first includes an update on research concerning the psychopharmacological treatment of depression. Physicians randomized to either algorithm group (CDSS-D or PPA-D) then receive training in the use of their respective algorithmic approaches by the study investigators. Finally, physicians randomized to CDSS-D are trained to previously established competence levels in the use of the software. Protocol details, as well as study procedures for providing technical support, are discussed during training. During the study, the principal investigator and other project management team members are available via weekly teleconferences to provide additional guidance in algorithm-compatible approaches to care.

Technical software support and algorithm specialists are available on-site when patient enrollment begins and remain until clinicians demonstrate proficiency in the use of the algorithm and software (CDSS-D and PPA-D). After start-up, support during CDSS-D patient visits is provided remotely from study headquarters via Web-based, Health Insurance Portability and Accountability Act (HIPAA)-compliant, secure, desktop sharing with audio streaming technology. This technology allows technical support personnel to monitor study patient visits in real time and to coach study clinicians in the use of the software as necessary.

2.4. Patient enrollment and treatment

The study employs broad inclusion and minimal exclusion criteria to reflect its hybrid efficacy-effectiveness nature, although patients must not be planning to move away from the area of the study clinic during the duration of their participation in the study. Consenting patients are recruited if they are 18 years and older with a diagnosis of major depressive disorder [34] (with or without psychotic symptoms) as verified by the Structured Clinical Interview for DSM-

IV (SCID) [35] and a 17-item Hamilton Rating Scale for Depression ($HRSD_{17}$) score of 14 or greater [36], and are being treated by a study-enrolled clinician. All patients who are being treated by participating physicians and who meet criteria and provide informed consent will be eligible to participate. The inclusion of all qualifying, consenting patients treated within a fixed period of time avoids selection biases of the kind that randomization is also designed to address.

Exclusionary criteria include: a) an Axis I diagnosis of bipolar disorder, somatization, anorexia nervosa, bulimia, or obsessive compulsive disorder; b) pregnancy and other medical conditions contraindicating antidepressant medications used in the first two stages of the algorithm (e.g., thyroid conditions with inadequate work-up, diagnosed seizure condition); c) clinical status requiring inpatient or day treatment at the time of study enrollment; d) current participation in any protocol-driven cognitive behavioral psychotherapy specifically targeted for MDD; e) the presence of substance dependence within the last 3 months; and f) illiteracy or the absence of a working telephone to use for follow-up assessments.

2.5. Summary of a treatment visit

During treatment visits in both algorithm groups (CDSS-D and PPA-D), the physician assesses five critical aspects of patient status, including: 1) symptom severity, 2) functional status, 3) side-effect severity, 4) global change from baseline, and 5) medication adherence [37].

In the computerized treatment group, progression through the software facilitates this assessment of clinical status prior to decision making related to medication prescription. Once the clinician collects information on symptom intensity, functional status, and side-effect burden as reported by the patient during the visit, the software incorporates this information into graphical displays charted across time.

For CDSS-D clinicians, the Computerized TMAP software (CompTMAP) offers suggestions about algorithm-compatible recommendations, formulated from current patient status information and the length of time the patient has been on a particular medication regimen. Clinicians can either agree with or override computer-generated recommendations, but if they override the software-generated recommendations, they must provide a rationale for their decision. The software also incorporates optional subroutines and algorithms for management of side effects, combined or augmented pharmacological treatments, and automated end-of-visit procedures such as prescription writing, next appointment cards, and progress notes.

As noted above, PPA-D clinicians also collect specific information on patient status at the beginning of the patient visit. However, in contrast to CompTMAP software-generated treatment recommendations provided at the point of care, clinicians in the PPA-D group use the more traditional paper-based treatment manual describing algorithm-compatible options for the next treatment step. Physicians in the UC-D group deliver treatment as they normally would, assess status as usual, and fill out routine clinical paperwork at each study patient visit.

2.6. Computer software

The PC-based CDSS-D software program [32] consists of separate domains responsible for user interaction, decision tree reasoning, and storage of clinical data. Written on a Microsoft Windows platform using Visual Basic programming language, the interface allows users to navigate through interactive buttons and pull-down menus that provide a user-friendly environment in which to work. The Visual Basic presentation is the only portion of the CDSS-D program visible to the user. Our previously developed clinical algorithms have been translated into a “rules engine” using a logical inference engine licensed by FairIsaac Software™. The Rules Engine operates behind the user interface and employs current and historical patient data as it formulates algorithm-compatible treatment options supplied to the physician via the user interface. All clinical information entered into the CDSS-D software application is stored in a SQL Server™ database. The database also stores user-specific data and the reference tables for medications and dosage. This computer platform can be connected with other software files (i.e., administrative databases) to allow the collection of data required for specialized services, such as billing or reporting [38,39]. A step-by-step description of progression through the software program is provided in Appendix 1, and diagrams of the five basic screens associated with the software are found in Figs. 2–6.

2.7. Data collection and acquisition

From the date of enrollment, patients are followed for a total of 12 months. Follow-up evaluations occur every 6 weeks from the date of the baseline physician visit and are conducted by trained, blinded raters.

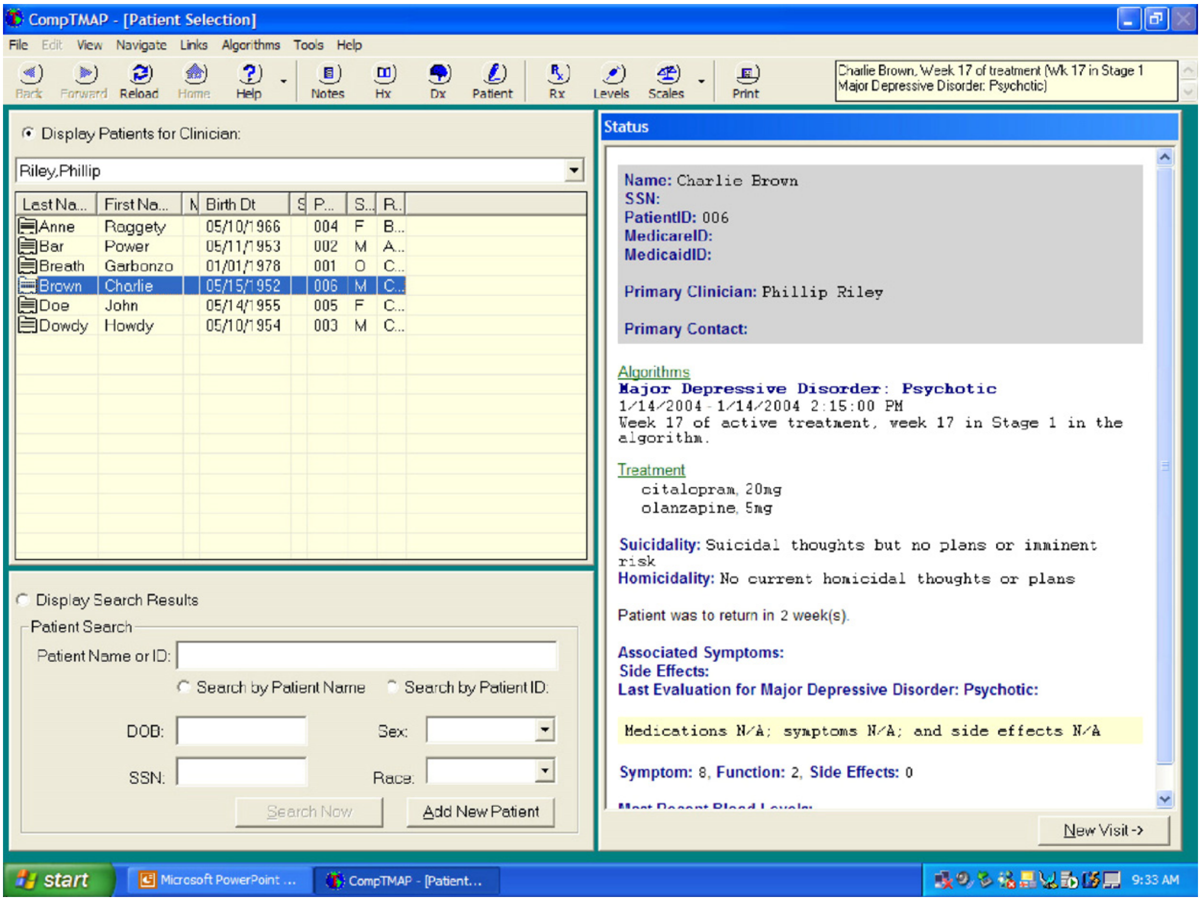

Fig. 2. CompTMAP — (Computerized Decisional Software): Screen #1.

Results of these 6-week evaluations are not shared with study clinicians, and therefore do not impact treatment decisions. Specific instrumentation and frequency of measurement are described below and shown in Table 1.

A number of factors were considered in determining the primary assessment instrument. For example, to measure depression severity, the Inventory of Depressive Symptomatology-Clinician-rated (IDS-C) was chosen as a primary measure of outcome because of its superior psychometric properties, greater sensitivity to changes in symptoms with treatment, and a greater range of symptom measurement [40]. The $HRSD_{17}$ was included as a secondary measure of symptom severity. Although at first glance it may appear redundant, the $HRSD_{17}$ will allow for direct comparisons with the large number of clinical trials that have used this measure to assess outcomes. In the case of measures of social functioning, multiple other factors were considered. While there are a large number of measures assessing social function, most of these measures have been developed for either disorders that cause specific physical limitations or are very global measures of function and are not depression specific. Thus, measures that would be most appropriate for a depressed patient population have not been developed. Moreover, our previous work has suggested a need for the assessment of function in multiple domains to obtain a full picture of the level of functional recovery. As a result, multiple measures of social function should allow us to better define dysfunction associated with depression [41].

1) Assessment of patient outcomes: Raters are blinded to treatment assignment at each data collection point (see Table 1). In addition, we have a well-developed telephone assessment system as described below (Jones AM, Flood A, Claassen C, Grannemann BD, Trivedi MH. Techniques for minimizing missing data and attrition: the use of telephone follow-up evaluations for assessing mental health status in a longitudinal study. Manuscript in preparation). Patient

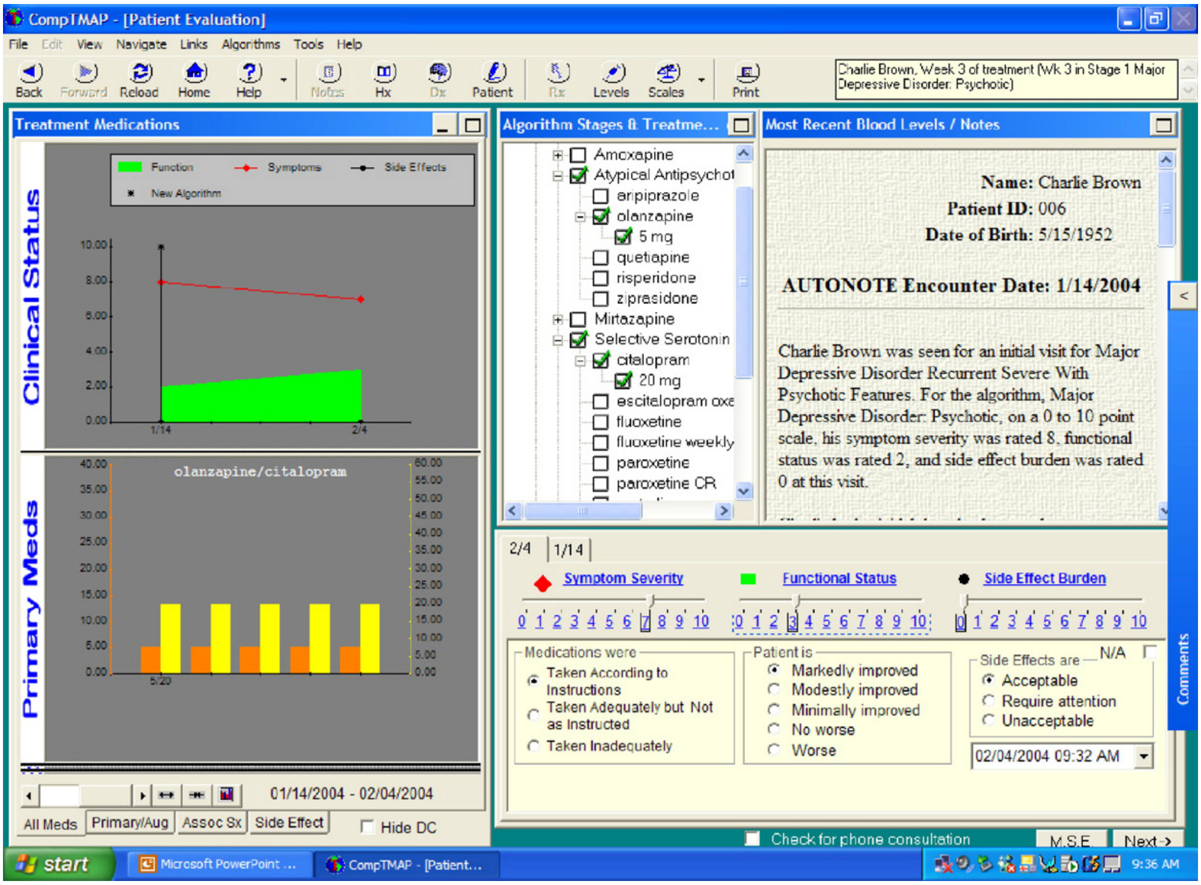

Fig. 3. CompTMAP — (Computerized Decisional Software): Screen #2.

outcome instrumentation has been selected specifically for its utility in assessing various domains that should be impacted by patient recovery, including:

a) Depressive symptoms:

Hamilton Rating Scale for Depression ($HRSD_{17}$ [36,42]): The $HRSD_{17}$ is considered the “gold standard” in clinician-administered rating scales designed to assess symptom severity in patients diagnosed with depression [43].

The 30-item Inventory of Depressive Symptomatology-Clinician-rated ($IDS\text{-}C_{30}$ [44–47]): The IDS is designed to measure the specific signs and symptoms of depression, including melancholic and atypical features, and was used as the main symptom outcome measure for the depression module of TMAP.

b) Cost of care:

Utilization and cost procedure: The Utilization and Cost Questionnaire (UAC-Q) [48–50] helps patients to list their health care providers and describe care use from each. Services are summarized into 3-month intervals, quantified into *units* (visit, days, encounters) and classified by *setting* (outpatient clinic, emergency room, inpatient facility, home, telephone, pharmacy, and jail), *diagnosis* (medical and surgical, psychiatric, addiction, rehabilitation, and dental), and *provider specialty* (medical, psychiatric, addiction, rehabilitation, and dental).

c) Quality of life:

Quality of Life, Enjoyment, and Satisfaction Questionnaire ($QLES\text{-}Q_{short\ form}$ [51]): The QLES-Q is designed to measure *satisfaction and enjoyment*, as opposed to function per se, in various domains of function: physical health, feelings, work, household duties, school/course work, leisure time activities, social relations, and general activities.

Work and Social Adjustment Scale (WSAS [52]): The WSAS is a 5-item, self-report measure designed to identify functional impairment that is attributed to an identified problem or condition.

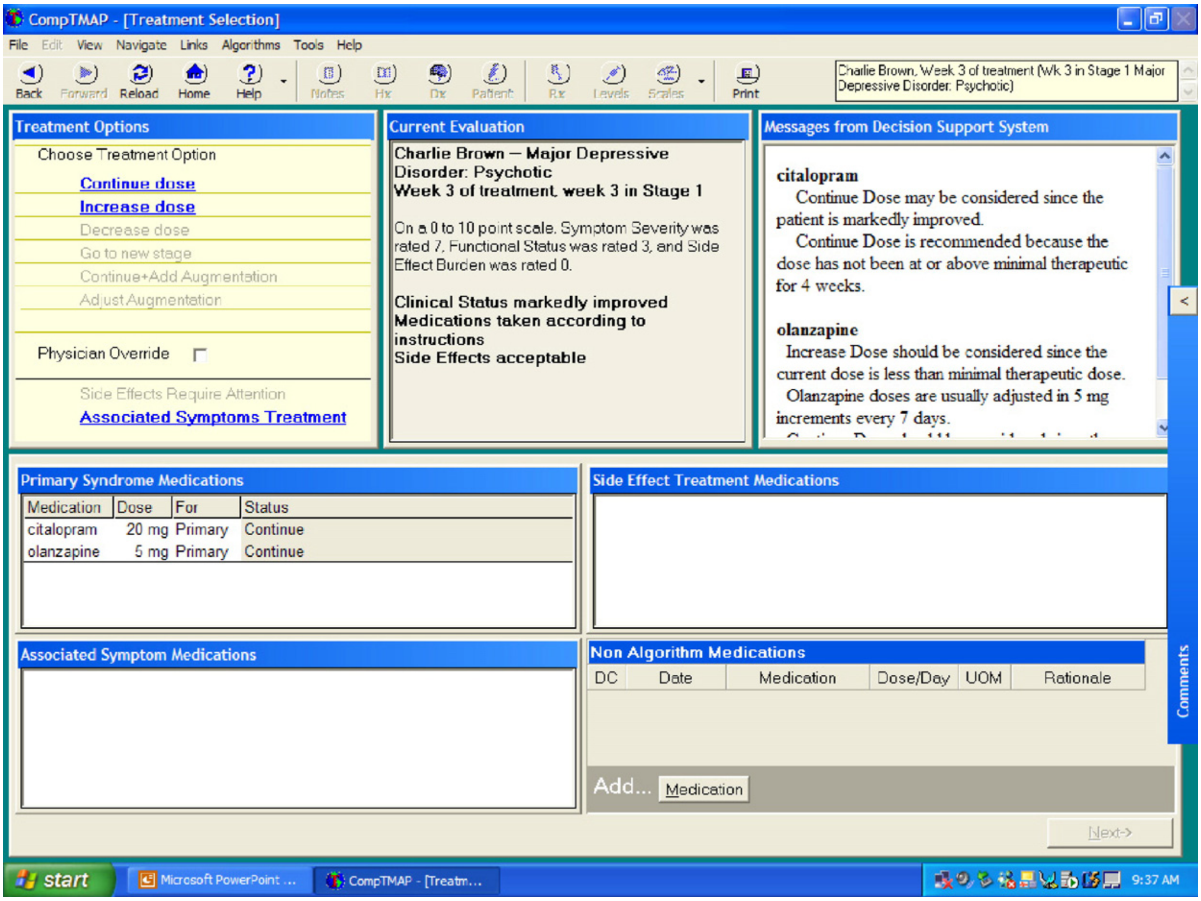

Fig. 4. CompTMAP — (Computerized Decisional Software): Screen #3.

Social Adjustment Scale-Self Report (SAS-SR [53]): The SAS-SR is a 54-item, self-report measure of instrumental and expressive role performance.

Short-Form Health Survey (SF-36 [54–56]): This measure assesses quality of life and general health [54–56], emphasizing the patient point of view. The measure contains eight scales measuring: physical functioning, physical role functioning, bodily pain, general health, vitality, social functioning, emotional role functioning, and mental health. The eight scale scores range from 0 to 100, with higher scores indicating better health.

Work and Productive Activity Impairment Index (WPAI [57]): The WPAI is a six-item scale that measures the number of work hours missed during the last 7 days, the number of hours actually worked in the last 7 days, and impairment while working or performing usual daily activities as a result of health problems.

d) Satisfaction with care:

Patient Perception of Benefits of Care (PPBC): The PPBC measures patient disposition to care-seeking behavior. TMAP MDD data ($N=545$) revealed PPBC was associated with clinical outcomes with a Cronbach's alpha of 92% with mean = 10, s.d. = 7, range 0 (believes appropriate care will be most helpful with activities of daily living) to 40 (believes appropriate care is ineffective), with 75% of subjects within the range from 5 to 15.

Computer Attitude Scale (CAS) and Computer Use Scale (CUS) are both designed to assess attitudes toward computers and their use in medical settings by either physicians or patients. The CAS is a measure of general attitudes towards computers.

e) Side effects:

Frequency, Intensity, and Burden of Side Effects Rating (FIBSER [58]): Developed for the National Institute of Mental Health (NIMH) multicenter clinical trial of Sequenced Treatment Alternatives to Relieve Depression

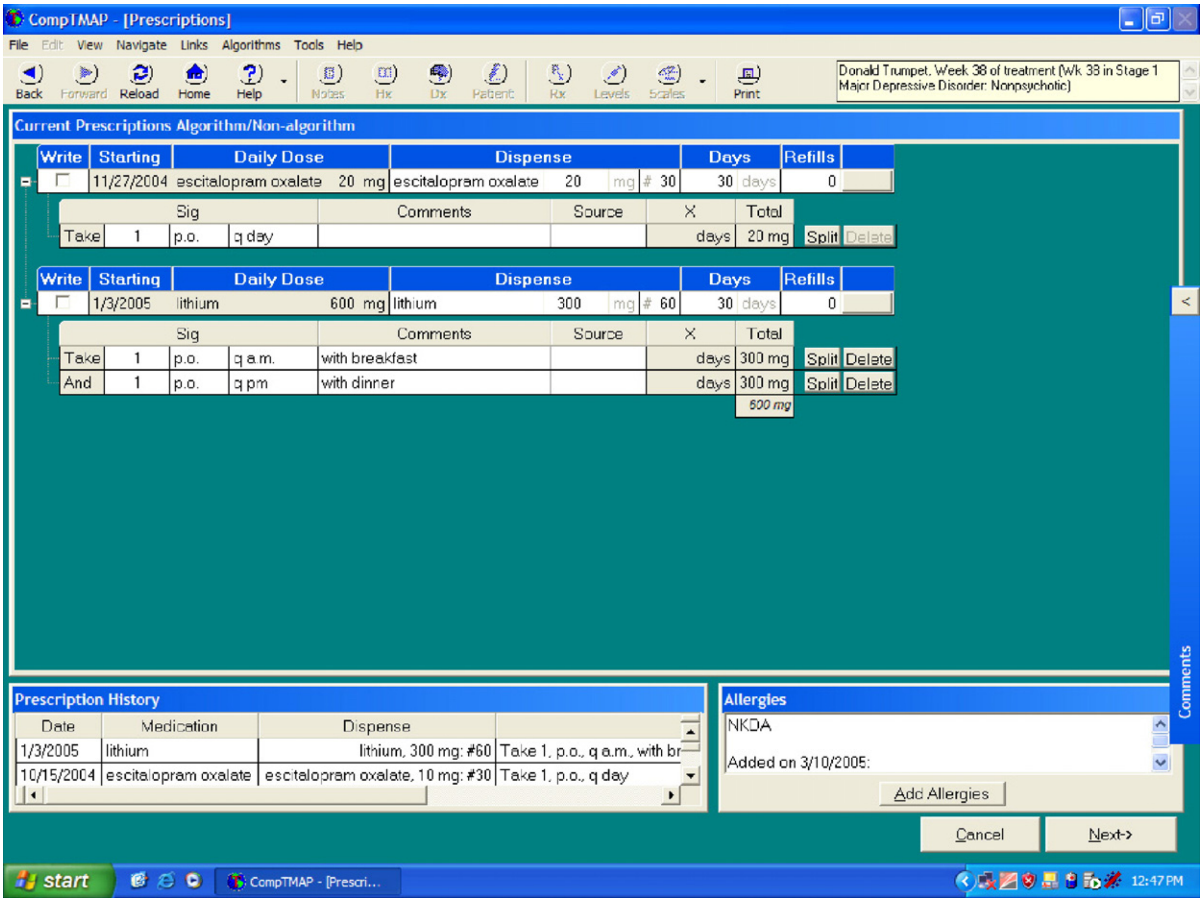

Fig. 5. CompTMAP — (Computerized Decisional Software): Screen #4.

(STAR*D) [59,60], the FIBSER utilizes global ratings on a Likert-type scale rated 0–6 for three scales — one rates the frequency, another intensity, and the third the overall burden or degree of interference in day-to-day activities and function due to side effects attributable specifically to the antidepressant treatment.

2) Assessment of physician treatment behavior: For both algorithm groups, we have developed a set of criteria that we use to monitor physician adherence to algorithm guidelines (Table 2). At the end of the study, for each patient's 12-month trial, a composite rating is generated quantifying the degree of physician adherence to the algorithm during treatment of that patient. The rating procedure assesses algorithm-specific "acts of omission" (appropriateness/inappropriateness of drug regimen, dose, dose increases, and frequency of provider contact); "acts of commission" (appropriateness/inappropriateness of medication changes for partial responders at less than 8 and 12 weeks); and completeness of information collected on side-effect burden, patient treatment adherence, and changes in symptoms. The physician's total score is a summation of scores in each of these areas divided by the total points available for each stage a patient has entered over the course of the trial.

For physicians in the usual care group, treatment data are collected from visit data forms completed at the time of the patient visit and include medication regimens and frequency of visit.

3) Assessment of physician demographics and attitudes: Demographic information, including gender, race, ethnicity, age, board certification, number of years employed, and patient/physician ratio is collected for all physicians at study entry. In order to gain a better understanding of factors that may influence the implementation of treatment algorithms, all study physicians (CDSS-D, PPA-D, and UC-D) also take surveys that measure 1) job satisfaction using the study-generated Physician Satisfaction Questionnaire, 2) attitudes towards computers in general using the study-generated CAS (see above), and 3) for the two algorithm-based treatment groups only, a

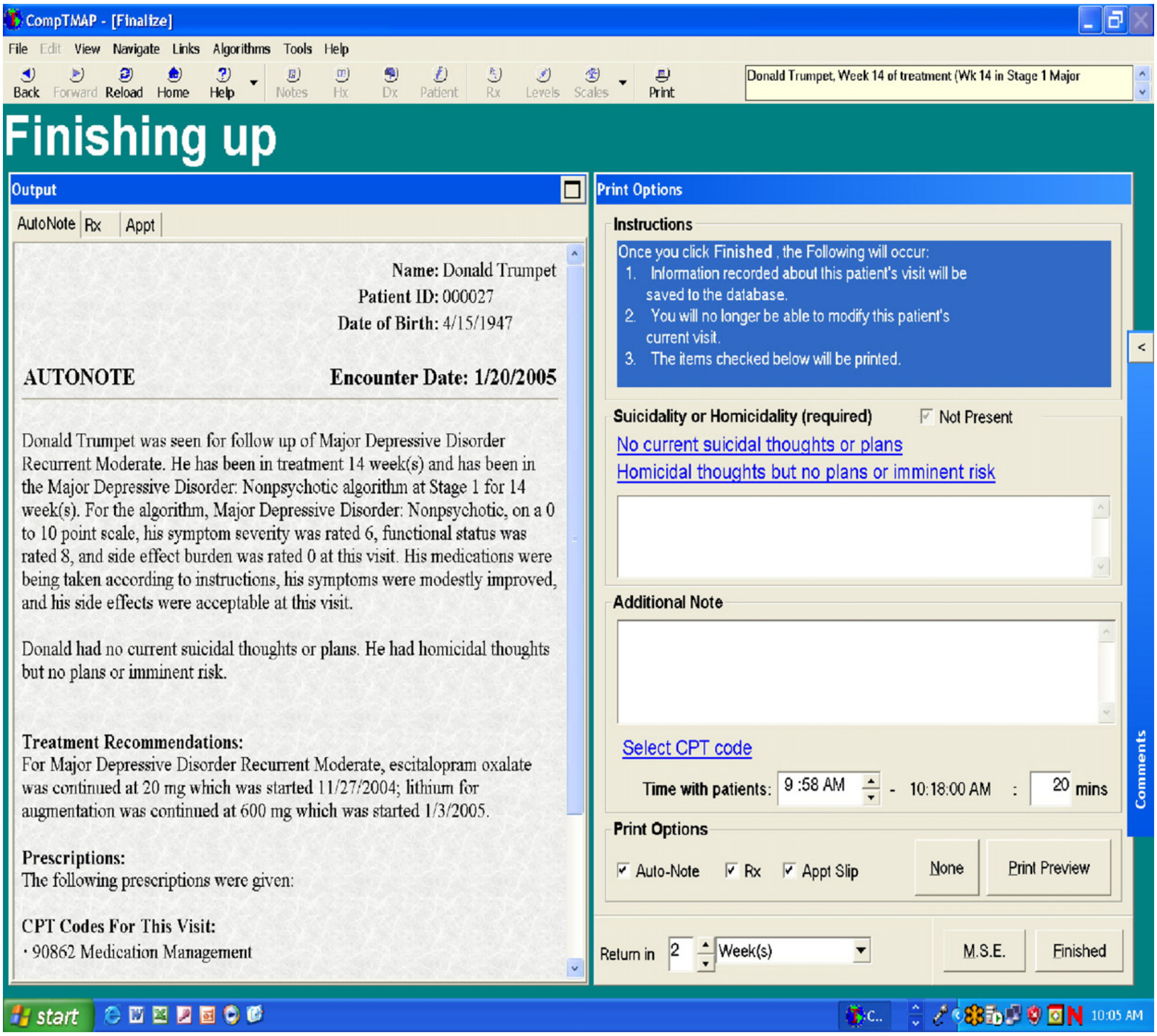

Fig. 6. CompTMAP — (Computerized Decisional Software): Screen #5.

study-generated survey intended to measure physician satisfaction with the algorithms (Ease of Use Survey). No information on the reliability or validity of these instruments currently exists; however, they were used in our previous TMAP study, so substantial data will be available to establish psychometric properties based on the two comparable samples.

4) Assessment of cost outcomes: To be consistent with US Public Health Service Panel on Cost Effectiveness in Medicine's recommendations [61–64] for applied mental health [65] and depression [66], direct costs of care are defined here as costs of patients accessing and using all health care services by type: care system, payer, provider specialty, setting, service type, and disorder. Direct costs will also be calculated consistent with the recommendations of the panel for mental health and depression. These include multiplying utilization by unit costs and summing overall services. Different unit cost schedules are planned, with sensitivity analyses designed to describe how cost estimates vary across different cost environments (external validity). Specifically, patient use of care will be measured by the Utilization and Cost (UAC) methodology [49,50]. UAC measures patient use of care from all provider sources by constructing hybrid files from provider records, administrative files, and patient self-reports. UAC begins with a patient questionnaire [49,59] listing health care providers and describing care use from each over the past 3 months. Providers are identified and located, and research associates send patient consent forms

Table 1
Project IMPACTS: patient assessment schedule by group

WEEK	0 Baseline			6			12			18			24			30			36			42			48			52 or end of trial		
GROUP<br>Grp 1 = CDSS-D<br>Grp 2 = PPA-D<br>Grp 3 = UC-D	1	2	3	1	2	3	1	2	3	1	2	3	1	2	3	1	2	3	1	2	3	1	2	3	1	2	3	1	2	3
**PT MEASURES**																														
Diagnostic Instrument																														
SCID-CV	•	•	•																											
**Symptom Severity**																														
HRS-D17-item	•	•	•	•	•	•	•	•	•	•	•	•	•	•	•	•	•	•	•	•	•	•	•	•	•	•	•	•	•	•
IDS-C30-item	•	•	•	•	•	•	•	•	•	•	•	•	•	•	•	•	•	•	•	•	•	•	•	•	•	•	•	•	•	•
**Utilization & Cost**																														
UAC-Q	•	•	•				•	•	•				•	•	•				•	•	•				•	•	•			
**Quality of Life/Function Measures**																														
QLES-Q short form	•	•	•	•	•	•	•	•	•	•	•	•	•	•	•	•	•	•	•	•	•	•	•	•	•	•	•	•	•	•
WSAS/SAS-SR	•	•	•	•	•	•	•	•	•	•	•	•	•	•	•	•	•	•	•	•	•	•	•	•	•	•	•	•	•	•
SF-36	•	•	•	•	•	•	•	•	•	•	•	•	•	•	•	•	•	•	•	•	•	•	•	•	•	•	•	•	•	•
WPAI	•	•	•	•	•	•	•	•	•	•	•	•	•	•	•	•	•	•	•	•	•	•	•	•	•	•	•	•	•	•
**Pt. Perception of Care**																														
CAS	•	•	•				•	•	•				•	•	•				•	•	•				•	•	•			
CUS	•						•						•						•						•			•		
PPBC	•	•	•				•	•	•				•	•	•				•	•	•				•	•	•			
**Side-Effect Burden**																														
FIBSER	•	•	•	•	•	•	•	•	•	•	•	•	•	•	•	•	•	•	•	•	•	•	•	•	•	•	•	•	•	•

and ask for faxed billing information (HCFA 1500, UB92) or data extracts describing patient use of care (event dates, diagnoses, CPT procedures, DRG stays). For nonreporting providers, information is obtained from third party data (e.g., state Medicaid, Medicare), or if not available, from patient UAC-Q responses.

To extend the generalizability of our findings, unit cost schedules are selected to reflect different cost environments, with results compared by schedule after using sensitivity analyses to determine a cost range. In a manner described elsewhere [48,67,68], public sector mental health unit cost accounts are computed for each current procedural terminology (CPT) code or diagnostic related groupings (DRG). To account for differences in cost between procedures done under PPA-D and CDSS-D, investigators will recalibrate published total facility weights for each CPT procedure to reflect the ratio of physician times required to perform the same procedure under CDSS-D and PPA-D [48]. Data will come from physician time sheets routinely kept by public sector mental health clinics for billing purposes, though sample estimates will be made periodically to ensure administrative data accuracy, with corrective action contingencies planned.

Finally, cost to charge ratios are computed based on production costs using public sector mental health cost accounts, and based on the nationwide Veteran Administration's Decision Support System and Cost Distribution Reports, exclusive of training and research activities, and adjusted to reflect patient mix [48]. Whenever provider records are unavailable, for each unit cost schedule, patient UAC-Q responses are translated directly into costs by multiplying reported volume by cost-weights calculated by comparing UAC-Q responses with provider-based cost estimates when both records exist [48].

2.8. Comparing direct costs with outcomes

Again, per Panel recommendations [61], we plan to compare changes in outcomes with changes in direct costs of care between CDSS-D, PPA-D, and UC-D with respect to cost-outcome using (1) *a ratio of differences*, based on Jerrell and Hu [69], which will determine how much additional outcome is produced for each additional health care dollar expended when one treatment alternative is compared to another; and (2) *a difference in ratios*, assessing the

Table 2
Project IMPACTS: criteria by which to monitor physician adherence to algorithm guidelines

Physician adherence measures			
Acts of omission	Yes = 1	N = 0	NA
Appropriate drug regimen	1	0	n/a
Appropriate dose	1	0	n/a
Dose increase if inadequate response	1	0	n/a
Adequate frequency for provider contact	1	0	n/a
Acts of commission			
Changing medication(s) if <50% response at <8 weeks	1	0	n/a
Changing medication(s) if <50% by week 12	1	0	n/a
Clinical information collected			
Side-effect burden	1	0	n/a
Patient treatment adherence	1	0	n/a
Symptom change	1	0	n/a

difference between treatment alternatives in the ratios of the average change in outcome per dollar of care expended. For the purposes of these analyses, outcomes will be measured by (1) average IDS-C_{30} scores and (2) depression-free days. Costs include annualized direct cost of care. Ratio of differences will be computed by constructing separate cost and outcome regression equations from treatment indicator predictor variables. Estimated simultaneously, the difference in ratios is computed by the ratio of coefficients to the treatment indicator predictor variables from the respective regressions. Standard errors will be calculated from bootstrap resampling methods [70,71] or from simulating distributions using estimated means and standard errors of the regression coefficients [72,73]. Differences in ratios will be calculated from two-stage least squares estimates of the coefficient to the [(predicted cost) × (treatment indicator)] interaction term entered, along with predicted treatment costs to the outcomes regression [74].

2.9. Sample size and power analysis

For continuous measures with our proposed sample of 540, we estimate we can detect in 80% of samples a difference of 5.9 points on the IDS-C_{30}, or 15% of baseline levels (mean = 38.6, s.e. = 13.68), based on a two-tailed *t*-test at $\alpha = .05$. The number of subjects was determined by *t*-test and then augmented by a design effect of $[1+\rho(B-1)]$, where "*B*" is the number of patients per physician and $\rho = .094$ estimated using our previous TMAP study baseline IDS-C_{30} assessment data. These power estimates are conservative because they do not take into account the proposed repeated measures. It should be noted that this 5.9-point difference is the minimum expected difference between groups, rather than the minimum clinically-detectible change in IDS-C_{30} scores expected from individual patients who respond to treatment. In fact, much larger change scores would be expected from individual patients who are successfully treated, and these will be offset by minimal change scores found among patients who experience no treatment response. As such, primary analyses are based on average differences between study groups, and not on the expected difference found among individual patients within each group.

For the categorical outcome of response (defined below), the proposed sample can detect a difference in response rates of 15% (e.g., 55% in PPA-D versus 70% in CDSS-D) in 90% of samples unadjusted, based on a two-tailed log rank test with $\alpha = 0.05$, and 30% lost to follow-up (PASS™ software) and 89% after the adjustment for clustering of patients within physicians. For the clustering adjustment, we compute a design effect as described above. Based on the design effect, we can compute an effective sample size using $N^{\text{eff}} = n/[1+\rho(B-1)]$ where "*B*" is the number of patients per physician, "*n*" is the total number of patients, and ρ is the intraclass correlation [75]. When ρ approaches 0, the effective sample size approaches the total 540 patients. When ρ approaches 1, the effective sample size approaches the total 45 clinicians.

The power of 89% is based on $\rho = 0.00297$ computed from our previous TMAP study using the estimator of ρ suggested by Donner [75]. If a larger clustering effect were found in the IMPACTS study, power would not be greatly affected. For $\rho = .020$, for example, the power is still .83.

2.9.1. Analytic plan

CDSS-D's impact on patient outcome will compare CDSS-D to PPA-D, CDSS-D to UC-D, and PPA-D to UC-D with respect to depressive symptom reduction (IDS-C_{30}), functioning, and quality of life. These outcomes will be treated as continuous measures, as a percent improvement from baseline values, and as dichotomous response to treatment indicated by $\geq 50\%$ improvement from baseline scores.

Assessing between-group differences on patient outcomes for both continuous and dichotomous outcomes, we will use a modified random-regression growth curve analysis [76,77]. This analytic approach takes into account randomization by physician, nesting of patients within physician, and nesting of repeated measures within patient; does not require fixed intervals between actual follow-up observations; measures how effects vary with time; allows for patients with missing observations to be included; and permits more flexible covariance structures for a better model fit including regression to the mean [78], heteroscedastic, and autocorrelated level-1 co-variance structures [79–81], and in addition can handle continuous, bivariate [82], and ordinal [83] data. Covariates include patient level (need, predisposing, and enabling [84,85]) and physician level factors (structure, e.g., years in practice, board certification, specialty, medical school affiliation [86]). Physician adherence rates will be incorporated into the analyses for CDSS-D's impact on patient outcome. The extent to which effectiveness of the algorithms (CDSS-D and PPA-D) are reduced when physician adherence is added to the above models will provide a measure of how physician adherence alone affects patient outcomes.

Physician adherence data will be directly assessed using logistic regression to compare the extent to which the CDSS-D and PPA-D physicians follow the treatments suggested by the algorithm. Differences in patient, physician, and site factors will be controlled and will match the factors in the patient outcome measures model.

Evaluation of the potential economic impact of CDSS-D will employ assessment of between-group differences (CDSS-D vs. PPA-D; CDSS-D vs. UC-D; PPA-D vs. UC-D) in utilization and costs computed using hierarchical linear and declining effects modeling [82,87]. Censored and bimodal distributions encountered with cost data will be modeled separately into use versus nonuse (logistic) and volume-of-use among users (continuous), as first explored by Duan [88], extended in Pohlmeier and Ulrich [89], and described econometrically in Maddala [90]. Skewed distributions in volume equations will be normalized with log or other appropriate transformation [91–93]. Predicted utilization will be obtained by multiplying the logit (probability of use) times the expected volume given use. Change in predicted values will determine between-group differences. Significance tests will be computed by bootstrapping samples with replacement [71].

For a difference in cost-outcome ratios we estimate by: $y_{dit} = b_0 + b_1 I_d + b_2 c_{dit} + b_3 (I_d c_{dit}) + w_d + v_{di} + u_{dit}$ where the t-distributed b_3 is the difference in cost outcome ratios between groups (CDSS-D vs. PPA-D; CDSS-D vs. UC-D; and PPA-D vs. UC-D). To avoid biased estimates resulting from actual costs correlated with random effects terms, we use an instrumental variable technique [94], replacing actual with predicted costs computed as described above. To compute the ratio of differences, we estimate outcomes: $y_{dit} = b_0 + b_1 I_d + b_2 t + b_3 (t I_d) + w_d + v_{di} + u_{dit}$ and cost: $c_{dit} = g_0 + g_1 I_d + g_2 t + g_3 (t I_d) + w_d + v_{di} + u_{dit}$ and calculate b_3 / g_3 as the change in outcomes per change in costs. Significance tests are again computed by bootstrapping samples with replacement [71].

2.10. Data and Safety Monitoring Board

The Data and Safety Monitoring Board (DSMB) for Project IMPACTS was created as an independent body charged with ensuring that study subject safety (both physicians and patients) is protected and that the scientific goals of the study are met. To support those goals, the DSMB reviews proposed amendments to the study protocol; monitors all serious adverse events (SAEs), dropouts, and nonserious adverse events; determines whether study procedures should be changed; and performs periodic review of the completeness and validity of data to be used for analysis.

Since treatment is not blinded except for certain assessment procedures, safety information for this study is reported to the DSMB in an unblinded manner, using study-assigned subject numbers. All SAEs are reported, regardless of any judgment about their relatedness to study medication regimens. Related information reported to the DSMB for each SAE includes information about the event and its outcome, dosing history for all study drugs, concomitant medications, the subject's medical history and current conditions, and all relevant laboratory data.

Given recent concerns about certain antidepressants, the DSMB reviews data that may reflect differences in between-group safety issues. At its quarterly meetings, the DSMB is provided with summaries of the numbers and rates of adverse events and dropouts by treatment group. These reports include types of events, severity, and treatment phase. The DSMB also receives an overview of the progress of patient intake and retention and adherence to clinic appointments.

2.11. Timeline

Project IMPACTS was implemented beginning in spring 2003. A project management team was formed and meets bimonthly to address strategic questions related to data fidelity and other study policy matters. A hands-on operations team meets weekly to address ongoing study logistics. Physician recruitment and training for the initial study site took place in July-August 2003, and physician support structures were put in place immediately thereafter. Patient enrollment began in August 2003 and is projected to be complete by October 2006 at an average enrollment of 14 new patients per month. The DSMB began meeting in winter 2004. Twenty months into enrollment, 898 potentially eligible public sector patients being treated for depressive symptoms had been contacted about study enrollment, resulting in 214 enrolled patients. In addition, a total of 705 telephone follow-up assessments have been completed out of a possible 710.

3. Discussion

While the need to find ways to facilitate reliable, low cost implementation of algorithms is great, any attempt to implement treatment algorithms in public sector care systems faces a host of challenges. Neither selection of outcome variables, criteria by which to assess effectiveness, nor appropriate statistical approaches are well developed. In Project IMPACTS, we have developed an innovative approach that separates measurement of the impact of algorithmic treatment on patient outcome from measurement of prescriber adherence to the new standards of care. Decreased levels of treatment adherence and increased disease severity, overcrowded and understaffed clinics, and inadequate physician support are all common in public sector care systems, and these conditions pose substantial challenges to treatment effectiveness research. Results of this study will contribute information about optimal, cost-effective ways to implement evidence-based treatment in such settings. If effective in this setting, the computerized decisional support approach evaluated in Project IMPACTS will likely be easily exportable to other busy practice settings.

To accomplish our goals, we have developed a number of new study strategies and instruments. Novel Project IMPACTS features include operationalization of a set of variables that quantify key implementation constructs (e.g., the perceived quality of prestudy algorithm training; the quality of physician responses to the algorithmic treatment approach — including the level of confidence in the algorithm's capacity to produce effective treatment outcomes, the nature and level of both patient and prescriber adherence to study-related procedures/treatment, characterization of system of care variables that can interfere with or enhance algorithm effectiveness, and techniques to establish the cost of various kinds of care). In addition, IMPACTS cost measures involve a state-of-the-art application of recently developed statistical approaches that set a high, but necessary, standard for study success. IMPACTS focus on study physicians as subjects is also novel and has prompted the development of a set of IRB-approved procedures for treating study physicians as human subjects.

IMPACTS design innovations include a comparison of two approaches for implementing a treatment algorithm in a specialty care setting (e.g., a paper-and-pencil format and a computerized algorithm platform). In addition, the randomization of study physicians into the three study arms, with study patients clustered by individual treatment provider, is likewise innovative. Our telephone-based assessment format has greatly decreased the potential for missing data (Jones AM, Flood A, Claassen C, Grannemann BD, Trivedi MH. Techniques for minimizing missing data and attrition: the use of telephone follow-up evaluations for assessing mental health status in a longitudinal study. Manuscript in preparation). Finally, the study implements measurement-based treatment algorithms into real life clinical settings, providing the opportunity to balance the dual research priorities of efficacy and effectiveness.

We considered alternatives to our three-group design. The two other study designs traditionally used to evaluate innovative treatment strategies (i.e., two-group and crossover) had the advantages of requiring fewer subjects or providing greater power with the same number of subjects. However, we believe that the shortcomings of each outweighed these benefits. The choice of controls in a two-group design would have limited the question that could be

answered. A two-group design comparing treatment outcomes between CDSS-D and UC-D controls would essentially be an effectiveness test of the CDSS-D against current practices, and using only CDSS-D and PPA-D would provide no way to estimate the effectiveness of the CDSS-D system over and above the true baseline of UC-D.

A crossover design was deemed impractical for this study question because training clinicians to use the CDSS-D requires extensive background in the use of algorithms that cannot later be undone. In addition, it does not provide sufficient information regarding the effects of the interventions on treatment outcome. Our three-group study design has two critical advantages: it allows us to directly test the impact of a CDSS against usual care, and it allows us to judge the relative effectiveness of a computerized, algorithm-based, decisional support system vs. a paper-and-pencil-based treatment algorithm.

A number of design factors were incorporated in order to ensure that the results would be generalizable to a wide variety of clinical and system level settings. These include randomization of the physicians and, through them, their patients, minimal exclusionary and broad inclusionary criteria, enrollment via a blinded evaluator to ensure unbiased selection of study patients by condition, and the use of multiple sites that serve a variety of patient populations. Our efforts to make the study generalizable are related to our goal to provide a test of a CDSS-D system that, if proven effective, can easily be implemented in a large number of systems, either as part of an electronic medical record (EMR) or as a stand-alone system that serves as an EMR, which incorporates decision support.

4. Summary and conclusions

If outcomes research is to successfully enhance quality of care, its scientific findings must be incorporated into clinical decision making [95]. Previous work clearly demonstrates that a well-designed treatment algorithm improves outcomes compared to usual care, but that improvements are modest, likely because adherence to the algorithm is low even under optimized conditions [96] and implementation of research is often delayed. These results support the need for improved delivery of algorithmic treatment recommendations. If found to be effective, computerized delivery systems, such as CompTMAP, will undoubtedly have broad applicability in terms of real-world practice.

Project IMPACTS addresses the critical question of how best to facilitate the utilization of algorithms during the treatment of depression, while also providing a second, large-scale prospective assessment of the effectiveness of a previously validated MDD treatment algorithm. Initial experience with this project demonstrates the feasibility of studies of this kind. Through this project, we hope to contribute research strategies that facilitate the integration of state-of-the-art treatment information into routine medical care settings.

Acknowledgements

This work is supported by R01 MH-164062-01A1, Computerized Decision Support System for Depression (CDSS-D), awarded through the National Institute of Mental Health, Madhukar H. Trivedi, M.D., Principal Investigator.

Appendix A. CompTMAP Overview:

CompTMAP, developed at the University of Texas Southwestern Medical Center, is a comprehensive computerized treatment algorithm for the treatment of psychiatric illnesses. The program is designed to be used by primary care physicians and psychiatrists. The guidelines and algorithms from which the computer decision support system was developed were from the Texas Medication Algorithm Project (TMAP), a large scale study designed to determine the clinical and economic value of using medication algorithms in the pharmacological management of patients with one of three major mental disorders: schizophrenia, bipolar disorder, and major depressive disorders.

CompTMAP is designed to provide instantaneous decision making tools during the patient visit and record clinical information obtained during the clinical interaction. Through the connection with the central server, it creates a large, up-to-date patient database that, over time, provides further outcome-based prescriptive feedback to the physician on an ongoing basis. Finally, evidence-based revisions in the algorithm can be provided to treating physicians from the central server without user intervention.

The computer acts as a comprehensive recording system in the following manner: it serves as a retrieval system for patient information, including assessment of clinical progress; provides treatment recommendations, including prescriptions, return to clinic, and treatment options; facilitates medical record keeping; and provides information for billing. The software provides graphic, easy-to-use interfaces, and standard commands to enhance the physician's ability to interact with the computer during patient contact. This timely interaction is intended to facilitate retrieval of past information, as well as processing of current data, thus hastening clinical decision making. New patient data will be automatically linked to other data-gathering systems, including demographic information and state reporting requirements. It can also interface with computerized patient self-assessment tools (IDS-SR, symptom checklists, etc.). It is user-friendly and can be integrated in routine clinical practice. Thus, the computer serves as a tool to help the physician monitor progress and determine treatment strategies and tactics.

CompTMAP can be loaded on any personal computer with the recommended system requirements. CompTMAP consists of three separate parts responsible for user interaction, decision tree reasoning, and storage of the clinical data. The relationships between these three parts are as follows:

- The user interface: CompTMAP is an interactive application written for Microsoft Windows platform and developed using Visual Basic programming language. Users can navigate through Web-like buttons that provide a user-friendly environment in which to work. It is the only application of CompTMAP program that is visible to the user.
- The rules engine: The clinical algorithms used by TMAP have been translated into specific "rules" by Drs. Trivedi and Kern and the Computer Information Services (CIS) developers and have been compiled into a knowledge base and implemented using the industry-standard logical inference engine licensed from FairIsaac. The rules engine application operates behind the user interface to apply TMAP algorithms to patient data in order to provide treatment options to the physician.
- CompTMAP database: All clinical information entered into the CompTMAP application is securely stored in the back-end SQL server. The database also stores user-specific data (for limiting access to clinical information), and the reference tables for medications and doses.

The clinician is supported in clinical decision(s) by using this interactive tool that analyzes the pertinent information about the patient that has been provided by the clinician and integrates the patient's information with the rules of the program, which is based on expert knowledge. For each visit, the clinician is asked to enter information about the patient's current clinical condition that is necessary for analysis by the treatment algorithm. The required information addresses three aspects of the patient's current status: (1) the patient's compliance (i.e., whether the medication(s) has been taken as directed and/or adequately); (2) the patient's response to treatment (i.e., whether the patient improved markedly, modestly, minimally, not at all, or the conditioned worsened); and (3) side-effect burden (i.e., whether side effects were acceptable, not acceptable, or not significant). Once this information is entered, the "rules engine" of the software is invoked. The rules engine analyzes the new information about the patient that was entered by the clinician along with several other factors, such as the medication(s) the patient is on, the dose level, the amount of time at that dose level, how long the patient has been on the current treatment, how many dose increases, and medication blood levels (if applicable). After analyzing the information, the computer program will offer the appropriate treatment options(s) with dose options. The program provides suggestions to assist the physician in treating with the primary and augmenting medications, and also provides choices for treatments for associated symptoms and side effects. Explanations and suggestions are provided in a decision support window on the same screen on which the treatment options are displayed.

To ensure physician autonomy, the physician can deviate from the algorithm recommendations at any time by clicking the physician "override" box and providing rationale. All possible medication options will then be enabled and the physician can make his or her selection.

Follow-up and preventive care: CompTMAP provides reminders to the doctor via screen prompts so that he or she does not overlook important considerations. For example, if a doctor prescribes a mood stabilizer (e.g., lithium, or some other medication requiring close monitoring of blood levels), he or she will be prompted to order blood levels for that medication. The program can also recommend and display in how many weeks the patient should return for a visit, based upon the patient's status and stage in the algorithm.

Computer physician order entry and error prevention: In the CompTMAP software program, the clinician initially chooses the psychotropic medication(s) and doses from pull-down menus of the algorithm on the Treatment Selection Screen. The selected medication(s) then appear on the Prescription Screen along with suggested route and frequency.

The clinician can choose to adjust the frequency and type in specific instructions in the comments section. The clinician clicks on the check box next to the medication the patient needs and the prescription will be printed for the patient. This includes medication choices for primary medications and augmenting medications, as well as treatments for associated symptoms and side effects.

Adverse drug event alert systems: The CompTMAP software program displays a warning box when medication errors are made. For example, if a physician tries to order two benzodiazepines, a warning box will display that notifies the physician that he/she has ordered two medications from the same family of medications. For medications that require a blood level to safely increase the dose, a warning box will display that notifies the physician that a blood level is necessary. If a physician tries to order two medications that should not be given together or two medications that should be given together with caution, a warning box will display notifying the physician of the potential problem. It will also alert the physician if medication blood levels are not in the therapeutic range.

Electronic documentation, record keeping, and information retrieval: All entries are automatically stored, providing electronic documentation and record keeping. In turn, this information is easily available to the physician at any time, providing access to complete patient information. Clinical status and prescription history are presented in easy-to-read graphs for each visit (Fig. 3). Additional information (such as patient demographics, blood level, symptom rating scales, complete progress notes) is also accessible by clicking on the toolbar at the top of the screen in any section of the application.

Automatic physician notes are created and recorded as a by-product of the physician's actions during a visit; additional notes can be written on the "slide note" that is available. The patient's progress is recorded throughout the course of care as progress notes and is also displayed graphically showing the patient's status (symptom severity, functional status, and side-effect burden) over time (Fig. 3). The medication choices are also recorded in the progress notes, prescription history, as well as graphically. The graphic display presents an "at a glance" recording of the patient's treatment and response over time. The patient's demographics, history, physician ratings, mental status examination, symptom scale assessments, and blood levels are a part of the record.

The application also provides safety-related reminders and documentation. For example, when a new medication is prescribed, the application prompts the physician to answer whether side effects and benefits of the new medication were explained in order to exit the visit. The automatic progress note documents that the physician explained the side effects and benefits if this question is answered "yes". Also, the physician must document the presence or absence of suicidal/homicidal ideation, plans, and intent. When homicidal or suicidal ideation is recorded, this information is presented in red at the next visit to alert the physician to reassess. A pull-down list of CPT codes or other billing codes that can be customized to the clinic is also available to assist in billing. Finally, paperwork burden is reduced because of electronic documentation and the ability of the software to be customized to link with internal pharmacy and billing systems.

The CDSS can be integrated into currently used clinic systems, interface to another computer application, import and export data and files, and have online access to a main database. The program provides links to the Texas Medication Algorithm Project's manuals and flow charts, the American Psychiatric Association Home Page and Clinical Practice Guidelines, and the CompTMAP manual.

Description of the use of CompTMAP: The computerized algorithm application begins with a *Sign-on Screen* where the user must enter his or her user name and password to access the program. The software utilizes role-related access security, meaning that different levels of access can be granted to individuals depending on their role in the clinic (i.e., only physicians would be granted access to progress through the algorithm or write prescriptions, but administrative personnel would have access to patient demographic information).

When a clinician signs on with his/her password, the first screen after the Sign-on Screen will be the *Patient Selection Screen* (Fig. 2) where a list of patients assigned to that particular clinician will be displayed under his/her name. To access a patient's record, the clinician simply clicks the patient's name to display a synopsis of the patient's care. The next screen is the *Diagnosis Screen* (for a new patient) and the Patient Evaluation Screen (Fig. 3)(for a returning patient). On the Diagnosis Screen a clinician can choose the Axis I, II, III, IV, and/or V diagnosis from a cascade of drop-down boxes. Multiple diagnoses can be listed in Axis I, II, or III. Once the specific algorithm and Axis I diagnosis are entered for a new patient, the *next* button will take the clinician to the Patient Evaluation Screen.

For a new patient, the *Patient Evaluation Screen* (Fig. 4) asks the physician to rate the patient's current level of symptom severity, functional status, and side effects (if applicable). A Mental Status Exam is available on this screen, as well as access to scales appropriate for that patient's diagnosis that can assist the clinician in determining the patient's status.

For a return visit, the Patient Evaluation Screen displays the patient's current stage in the algorithm, current medications (marked with green checks) and discontinued medications (marked with red x's). The most current progress notes and blood levels are displayed, as well as graphs recording the patient's clinical progress (symptom severity, functional status, and side effects) and medications prescribed over time. The clinician's evaluation of the patient, if it is a new patient, simply records in the database for future visits. However, if it is a return visit, the physician is asked to enter information about the patient's current clinical condition that is necessary for analysis by the treatment algorithm. The required information addresses three aspects of the patient's current status: (1) the patient's compliance (i.e., whether the medication(s) has been taken as directed and/or adequately); (2) the patient's response to treatment (i.e., whether the patient improved markedly, modestly, minimally, not at all, or the conditioned worsened); and (3) side-effect burden (i.e., whether side effects were acceptable, not acceptable, or not significant). Once this information is entered, the "rules engine" of the software is invoked. The rules engine analyzes the new information about the patient that was entered by the physician along with several other factors, such as how long the patient has been on the current treatment, what medication (s) the patient is on, the current dose level, the amount of time at that dose level, the number of dose increases, whether the patient is being augmented, and medication blood levels (if applicable). After analyzing the information, the computer program will offer the appropriate treatment options(s) with dose options on the next screen, the Treatment Selection Screen.

For a new patient, the treatment options are displayed for the clinician to choose from on the *Treatment Selection Screen* (Fig. 4). To select the recommended option, the clinician simply clicks on the choice and selects the desired medication and dose. The program provides suggestions to assist the physician in treating with the primary and augmenting medications, and also provides choices for treatments for associated symptoms and side effects. The clinician can click on *side effects require attention* or *associated symptoms treatment* to select the appropriate management of any symptoms. Nonalgorithm medications can be listed on the screen.

For the return visit, the *Treatment Selections Screen* (Fig. 4) will provide suggestions, such as Continue Dose, Increase Dose, Decrease Dose, Continue and Augment, or Go to Next Stage. Only the blue and underlined options are enabled. Explanations and suggestions are provided in a decision support window on the same screen on which the treatment options are displayed. The physician may override generated suggestions by clicking the *Physician Override* box, which prompts the physician to record a rationale for the override and then allows the physician to select the preferred intervention.

The Treatment Selection Screen provides reminders to the doctor via screen prompts so that he or she does not overlook important considerations. For example, if a doctor prescribes oxcarbazepine, he or she will be prompted to order sodium levels.

Once selected, the chosen medication(s) will move to the various treatment boxes on the bottom half of the screen and the physician can then click "*next*" to go to the *Prescription Screen* (Fig. 5). The selected medication(s) then appear on the Prescription Screen along with suggested route and frequency. This includes medication choices for primary medications and augmenting medications, as well as treatments for associated symptoms and side effects, and nonalgorithm medications. The physician can choose to adjust the frequency and type in specific instructions in the comments section. The physician clicks on the check box next to the medication the patient needs and the prescription will be printed for the patient. The clinician can also select whether the medication is prescribed or a sample was given.

Finally, the physician can finish the patient's visit by clicking "*next*" to go to the *Finishing up Screen* (Fig. 6). On this screen, the physician will see the computer generated progress note, which summarizes the current visit by incorporating the clinical ratings and prescriptions given. On this screen, the application also provides safety-related reminders and documentation. For example, when a new medication is prescribed, the application prompts the physician to answer whether side effects and benefits of the new medication were explained in order to exit the visit. The automatic progress note documents that the physician explained the side effects and benefits if this question is answered "yes". At this point, the physician can record any additional notes to be incorporated into the progress notes, select a CPT code or some other billing code from a drop-down menu, record the time spent with the patient (this will automatically record, and can be adjusted), assess suicidal and homicidal ideation, and indicate when the patient should return. Prescriptions and appointment slips for the patient and progress notes for filing in a physical chart are then printed out once the note is finished.

Additional screens in CDSS-D, which can be selected during the visit (or anytime) by clicking the appropriate button on the tool bar include: Blood Level Screen (for recording and viewing blood levels when necessary), Scales

Screen (which charts the scores of standardized rating scales), Notes Screen (a compilation of the progress notes of all visits), and History Screen (familial and psychosocial history plus a mental status exam completed at intake).

References

[1] Depression Guideline Panel. Clinical Practice Guideline. Depression in Primary Care: Volume 1: Detection and Diagnosis. Publication No. 93-0550. Washington, DC: U.S. Department of Health and Human Services–Agency for Health Care Policy and Research; 1993.
[2] Depression Guideline Panel. Depression in Primary Care: Volume 2: Treatment of Major Depression. Publication No. 93-0551. Washington, DC: U.S. Department of Health and Human Services–Agency for Health Care Policy and Research; 1993.
[3] Wells KB, Stewart A, Hays RD, et al. The functioning and well being of depressed patients: results from the Medical Outcomes Study. JAMA 1989;262:914–9.
[4] Rush AJ, Trivedi MH. Treating depression to remission. Psychiatr Ann 1995;25:704–5.
[5] Hirschfeld RM, Keller MB, Panico S, et al. The National Depressive and Manic–Depressive Association consensus statement on the undertreatment of depression. JAMA 1997;277:333–40.
[6] Kessler RC, Berglund P, Demler O, et al. The epidemiology of major depressive disorder: results from the National Comorbidity Survey Replication. JAMA 2003;289(23):3095–105.
[7] Adli M, Rush AJ, Moller HJ, Bauer M. Algorithms for optimizing the treatment of depression: making the right decision at the right time. Pharmacopsychiatry 2003;36(Suppl 3):222–9.
[8] Crismon MLT, Trivedi MH, Pigott TA, et al. The Texas Medication Algorithm Project: report of the Texas Consensus Conference Panel on medication treatment of major depressive disorder. J Clin Psychiatry 1999;60:142–56.
[9] Schoenbaum SC, Gottlieb LK. Algorithm-based improvement of clinical quality. Br Med J 1990;301:1374–6.
[10] Francis A, Docherty JR, Kahn DA. The expert consensus guideline series, treatment of schizophrenia. J Clin Psychiatry 1996;57(Suppl 12B):1–58.
[11] Trivedi MH, Rush AJ, Crismon ML, et al. Clinical results for patients with major depressive disorder in the Texas Medication Algorithm Project. Arch Gen Psychiatry 2004;61(7):669–80.
[12] Dennehy EB, Suppes T, Rush AJ, et al. Does provider adherence to a treatment guideline change clinical outcomes for patients with bipolar disorder? Results from the Texas Medication Algorithm Project. Psychol Med 2005;35(12):1695–706.
[13] Headrick LA, Speroff T, Pelecanos HI, Cebul RD. Efforts to improve compliance with the National Cholesterol Education Program guidelines. Arch Intern Med 1992;152:2490–6.
[14] Margolis CZ, Warshawsky SS, Goldman L, et al. Computerized algorithms and pediatricians' management of common problems in a community clinic. Acad Med 1992;67:282–4.
[15] Bettinger TL, Crismon ML, Trivedi MH, Grannemann B, Shon SP. Clinicians' adherence to an algorithm for pharmacotherapy of depression in the Texas public mental health sector. Psychiatr Serv 2004;55(6):703–5.
[16] Gorton TA, Cranford CO, Golden WE, Walls AC, Pawelak JE. Primary care physician's response to dissemination of practice guidelines. Arch Fam Med 1995;4:135–42.
[17] Field MJ, Lohr KN. Clinical Practice Guidelines. Washington, DC: National Academy Press; 1990.
[18] Davis DA, Thomson MA, Oxman AD, Haynes RB. Changing physician performance. A systematic review of the effect of continuing medical education strategies. JAMA 1995;274(3):700–5.
[19] Tierney WM, Hui SL, McDonald CJ. Delayed feedback of physician performance versus immediate reminders to perform preventive care. Effects on physician compliance. Med Care 1986;24(8):659–66.
[20] Rush AJ, Crismon ML, Kashner TM, et al. Texas Medication Algorithm Project, phase 3 (TMAP-3): rationale and study design. J Clin Psychiatry 2003;64(4):357–69.
[21] Fava M, Rush AJ, Trivedi MH, et al. Background and rationale for the sequenced treatment alternatives to relieve depression (STAR*D) study. Psychiatr Clin North Am 2003;26(2):457–94 x.
[22] Rush AJ, Fava M, Wisniewski SR, et al. Sequenced Treatment Alternatives to Relieve Depression (STAR*D): rationale and design. Control Clin Trials 2004;25(1):119–42.
[23] Trivedi MH, Rush AJ, Wisniewski SR, et al. Evaluation of outcomes with citalopram for depression using measurement-based care in STAR*D: implications for clinical practice. Am J Psychiatry 2006;163(1):28–40.
[24] Trivedi MH, Kern JK, Grannemann BD, Altshuler KZ, Sunderajan P. A computerized clinical decision support system as a means of implementing depression guidelines. Psychiatr Serv 2004;55(8):879–85.
[25] Trivedi MH, Rush AJ, Gaynes BN, et al. 2006. Maximizing the adequacy of medication treatment in controlled trials and clinical practice: STAR*D measurement-based care. Neuropsychopharmacology; submitted for publication.
[26] Avorn J, Soumerai SB. Use of a computer-based Medicaid drug data to analyze and correct inappropriate medication use. J Med Syst 1982;6 (4):377–86.
[27] Ebell MH, Hale W, Buchanan JE, Dake P. Hand-held computers for family physicians. J Fam Pract 1995;41(4):385–92.
[28] Gross PA. Implementing evidence-based recommendations for health care: a roundtable comparing European and American experiences. Jt Comm J Qual Improv 2000;26(9):547–53.
[29] Hunt DL, Haynes RB, Hanna SE, Smith K. Effects of computer-based clinical decision support systems on physician performance and patient outcomes: a systematic review. JAMA 1998;280:1339–46.
[30] Linnarsson R. Decision support for drug prescription integrated with computer-based patient records in primary care. Med Inform (Lond) 1993;18(2):131–42.

[31] Tierney WM, Overhage JM, Takesue BY, et al. Computerizing guidelines to improve care and patient outcomes: the example of heart failure. J Am Med Inform Assoc 1995;2:316–22.
[32] Trivedi MH, Kern JK, Grannemann B, Altshuler KZ, Sunderajan P. A computerized clinical decision support system as a means of implementing depression guidelines. Psychiatr Serv 2004;55(8):879–85.
[33] Trivedi MH, DeBattista C, Fawcett J, et al. Developing treatment algorithms for unipolar depression in Cyberspace: International Psychopharmacology Algorithm Project (IPAP). Psychopharmacol Bull 1998;34:355–9.
[34] American Psychiatric Association. Diagnostic and Statistical Manual of Mental Disorders. 4th ed. Washington DC: American Psychiatric Press; 2000. Text Revision.
[35] First MB, Spitzer RL, Gibbon M, Williams JBW. Structured Clinical Interview for DSM-IV Axis I Disorders, Clinician Version (SCID-1-CV). New York, NY: Biometrics Research Department, New York State Psychiatric Institute, Department of Psychiatry, Columbia University; 1995.
[36] Hamilton M. A rating scale for depression. J Neurol Neurosurg Psychiatry 1960;12:56–62.
[37] DeBattista C, Trivedi MH, Kern JK, Lembke A. The status of evidence-based guidelines and algorithms in the treatment of depression. Psychiatr Ann 2002;32:658–63.
[38] Trivedi MH, Kern JK, Baker SM, Altshuler KZ. Computerized medication algorithms and decision support systems in major psychiatric disorders. J Psychiatr Pract 2000;6:237–46.
[39] Trivedi MH, Kern JK, Voegtle T, Baker SM, Altshuler KZ. Computerized medication algorithms in behavioral health care. In: Dewan NA, Lorenzi N, Riley R, Bhattcharya SR, editors. Behavioral Health Care Informatics. Inc, New York, NY: Springer-Verlag; 2001.
[40] Rush AJ, Trivedi MH, Carmody TJ, et al. Self-reported depressive symptom measures: sensitivity to detecting change in a randomized, controlled trial of chronically depressed, nonpsychotic outpatients. Neuropsychopharmacology 2005;30(2):405–16.
[41] Trivedi MH, Rush AJ, Wisniewski SR, et al. Factors associated with health-related quality of life among outpatients with major depressive disorder: a STAR*D report. J Clin Psychiatry 2006;67(2):185–95.
[42] Hamilton M. Development of a rating scale for primary depressive illness. Br J Soc Clin Psychol 1967;6:278–96.
[43] Schwab JJ, Bialow MR, Holzer CE. A comparison of two rating scales for depression. J Clin Psychol 1967;23:94–6.
[44] Rush AJ, Giles DE, Schesser MA, et al. The Inventory of Depressive Symptomatology (IDS): preliminary findings. Psychiatr Res 1986;18:65–87.
[45] Rush AJ, Gullion CM, Basco MR, Jarrett RB, Trivedi MH. The Inventory of Depressive Symptomatology (IDS): psychometric properties. Psychol Med 1996;26:477–86.
[46] Rush AJ, Carmody TJ, Reimitz PE. The Inventory of Depressive Symptomatology (IDS): clinician (IDS-C) and self-report (IDS-SR) ratings of depressive symptoms. Int J Methods Psychiatr Res 2000;9:45–59.
[47] Trivedi MH, Rush AJ, Ibrahim HM, et al. The Inventory of Depressive Symptomatology, Clinician Rating (IDS-C) and Self-Report (IDS-SR), and the Quick Inventory of Depressive Symptomatology, Clinician Rating (QIDS-C) and Self-Report (QIDS-SR) in public sector patients with mood disorders: a psychometric evaluation. Psychol Med 2004;34(1):73–82.
[48] Kashner TM, Rush AJ, Altshuler KZ. Measuring costs of guideline-driven mental health care: the Texas Medication Algorithm Project. J Ment Health Policy Econ 1999;2:111–21.
[49] Kashner TM, Wicker A. Utilization and Cost Methodology: Instructions and Manual. Dallas, TX; 1999.
[50] Kashner TM, Suppes T, Rush AJ, Altshuler KZ. Measuring use of outpatient care among mentally ill individuals: a comparison of self-reports and provider records. Eval Program Plann 1999:22.
[51] Endicott J, Nee J, Harrison W, Blumenthal R. Quality of Life Enjoyment and Satisfaction Questionnaire: a new measure. Psychopharmacol Bull 1993;29:321–6.
[52] Mundt JC, Marks IM, Shear MK, Greist JH. The Work and Social Adjustment Scale: a simple measure of impairment in functioning. Br J Psychiatry 2002;180:461–4.
[53] Weissman MM, Prusoff BA, Thomson MA, Harding PS, Myer JK. Social adjustment by self-report in a community sample and psychiatric outpatients. J Nerv Ment Dis 1978;166:317–26.
[54] Ware JE, Snow KK, Kosinski M, Gandek B. SF-36 Health Survey: Manual and Interpretation Guide. Boston, MA: The Health Institute, New England Medical Center; 1993.
[55] Ware JE. SF-36 physical and mental health summary scales: a user's guide. Version 2. Quality Metrics, Inc.; 1997.
[56] Ware JE. Conceptualization and measurement of quality of life: comments on an evolving field. Arch Phys Med Rehabil 2003;84(Suppl 2):43–51.
[57] Reilly MC, Zbrozek AS, Dukes EM. The validity and reproducibility of a work productivity and activity impairment instrument. PharmacoEconomics 1993;4:353–65.
[58] Wisniewski SR, Rush AJ, Balasubramani GK, Trivedi MH, Nierenberg AA. Self-rated global measure of the frequency, intensity, and burden of side effects. J Psychiatr Pract 2006;12(2):71–9.
[59] Rush AJ, Fava M, Wisniewski SR, et al. Sequenced treatment alternatives to relieve depression (STAR*D): rationale and design. Control Clin Trials 2004;25(1):119–42.
[60] Fava M, Rush AJ, Trivedi MH, et al. Background and rationale for the Sequenced Treatment Alternatives to Relieve Depression (STAR*D) study. Psychiatr Clin North Am 2003;26(2):457–94.
[61] Gold MR, Siegel JE, Russell LB, Weinstein MC. Cost-Effectiveness in Health and Medicine. New York, NY: Oxford University Press; 1996.
[62] Weinstein MC, Siegel JE, Gold MR, Kamlet MS, Russell LB. Recommendations of the panel on cost-effectiveness in health and medicine. JAMA 1996;276:1253–8.
[63] Russell LB, Gold MR, Siegel JE, Daniels N, Weinstein MC. The role of cost-effectiveness analysis in health and medicine. JAMA 1996;276:1172–7.
[64] Siegel JE, Weinstein MC, Russell LB, Gold MR. Recommendations for reporting cost-effectiveness analyses. JAMA 1996;276:1339–41.
[65] Hargreaves WA, Shumway M, Hu T-W, Cuffel B. Cost-Outcomes Methods for Mental Health. San Diego, CA: Academic Press; 1998.

[66] Lave JR, Frank RG, Schulberg HC, Kamlet MS. Cost-effectiveness of treatments for major depression in primary care patients. Arch Gen Psychiatry 1998;55:645–51.
[67] Drummond MF, O'Brien BJ, Stoddard GL, Torrance GW. Cost effectiveness of thrombolytic therapy with streptokinase in elderly patents with suspected acute myocardial infarction. N Engl J Med 1992;327:7–13.
[68] Menke TJ, Homan RH, Kashner TM. Issues in determining costs in the Department of Veterans Affairs. Med Care 1999;37:AS18–26.
[69] Jerrell JM, Hu T-W. Cost-effectiveness of intensive clinical and case management compared with an existing system of care. Inquiry 1989;26:224–34.
[70] Efron B. The jackknife, the bootstrap and other resampling methods; 1982.
[71] Efron B, Tibshirani RJ. An Introduction to the Bootstrap. New York, NY: Chapman Hall; 1993.
[72] Mullahy J, Manning WG. Statistical issues in cost effectiveness analysis. In: Sloan F, editor. Valuing Health Care: Costs, Benefits, and Effectiveness of Pharmaceuticals and Other Medical Technologies. New York, NY: Cambridge University Press; 1994. p. 149–84.
[73] Manning WG, Fryback DG, Weinstein MC. Reflecting uncertainty in cost-effectiveness analysis. In: Gold MR, Siegel JE, Russell LB, Weinstein MC, editors. Cost-Effectiveness in Health and Medicine. New York, NY: Oxford University Press; 1996. p. 247–76.
[74] Siegel JE, Laska E, Meisner M. Statistical methods for cost-effectiveness analyses. Control Clin Trials 1996;17:387–404.
[75] Donner A, Birkett NJ, Buck C. Randomization by clusters: sample size requirements and analysis. Am J Epidemiol 1981;114:906–14.
[76] Gibbons RD, Hedeker D, Elkin I, et al. Some conceptual and statistical issues in analysis of longitudinal psychiatric data: application to the NIMH Treatment of Depression Collaborative Research Program Dataset. Arch Gen Psychiatry 1993;50:739–50.
[77] Jennrich RI, Schluchter MD. Unbalanced repeated measures models with structured covariance matrices. Biometrics 1986;42:805–20.
[78] Berndt ER, Finkelstein SN, Greenberg PE, et al. Workplace performance effects from chronic depression and its treatment. J Health Econ 1998;17:511–35.
[79] Louis TA, Spiro AI. Fitting First Order Auto-regressive Models with Covariates (Technical Report). Department of Biostatistics, Cambridge, MA: Harvard University School of Public Health; 1984.
[80] Hedeker D. 1989. Random Regression Models with Autocorrelated Errors. [Doctoral dissertation]. Chicago, IL: Academic Department: University of Chicago.
[81] Chi EM, Reinsel GC. Models of longitudinal data with random effects and AR(1) errors. J Am Stat Assoc 1989;84:452–9.
[82] Byrk AS, Raudenbush SW. Hierarchical Linear Models. Newbury Park, CA: Sage Publications; 1992.
[83] Hedeker D, Gibbons RD. A random-effects ordinal regression model for multilevel analysis. Biometrics 1994;50:933–44.
[84] Aday LA. The utilization of health services: indices and correlates. A Research Bibliography. DHEW Pub No. (HSM) 73-3003. Washington, DC: US Government Printing Office; 1972.
[85] Andersen R. A behavioral model of families' use of health services. Research Series, vol. 25. Chicago, IL: University of Chicago; 1968.
[86] Donabedian A. Explorations in Quality Assessment and Monitoring, Vols, I, II, and III. Ann Arbor, MI: Health Administration Press; 1985.
[87] Claassen CA, Kashner TM, Gilfillan SK, Larkin GL, Rush AJ. Psychiatric emergency service use after implementation of managed care in a public mental health system. Psychiatr Serv 2005;6(56):691–8.
[88] Duan N, Manning WG, Morris CN, Newhouse JP. A comparison of alternative models for the demand for medical care. J Bus Econ Stat 1983;1:115–26.
[89] Pohlmeier W, Ulrich V. An econometric model of the two-part process in the demand for health. J Hum Resour 1995;30:339–61.
[90] Maddala GS. Limited-dependent and qualitative variables in econometrics. New York, NY: Cambridge University Press; 1988.
[91] Theil H. Principles of Econometrics. New York, NY: Wiley and Sons; 1971.
[92] Kmenta J. Elements of econometrics. New York, NY: MacMillan; 1973.
[93] Amemiya T. Advanced Econometrics. Cambridge, MA: Harvard University Press; 1985.
[94] Heckman J. Instrumental variables: a study of implicit behavioral assumptions used in making program evaluations. J Hum Resour 1997;32:441–62.
[95] Donabedian A. Benefits in medical care programs. Cambridge, MA: Harvard University Press; 1976.
[96] Rush AJ, Trivedi MH, Carmody TJ, et al. One-year clinical outcomes of depressed public sector outpatients: a benchmark for subsequent studies. Biol Psychiatry 2004;56:46–53.
