# Supplementary material for: Barriers to implementation of a computerized decision support system for depression: an observational report on lessons learned in "real world" clinical settings
Source: BMC Med Inform Decis Mak. 2009 Jan 21;9:6. doi: 10.1186/1472-6947-9-6 (PMC2639574; doi:10.1186/1472-6947-9-6)
Supplement: Additional file 2 — CDSS Surveys. Algorithm Evaluation Questionnaire: Software Application Version. Part I: Ease of Use [file 1472-6947-9-6-S2.pdf]

Algorithm Evaluation Questionnaire: Software Application Version
Part I: Ease of Use

Directions: We are interested in your opinion on the ease of use of this software program. Please circle the number that corresponds with your opinion on this software program.

1. Overall how easy was the software program to use?

very difficult — difficult — easy — very easy

|--------|-------|--------|-------|--------|-------|
1 2 3 4 5 6 7

2. Was it easy to find what you were looking for on the screens?

very difficult — difficult — easy — very easy

|--------|-------|--------|-------|--------|-------|
1 2 3 4 5 6 7

3. Did the sequence of the screens follow a logical order?

not at all logical — slightly logical — mostly logical — very logical

|--------|-------|--------|-------|--------|-------|
1 2 3 4 5 6 7

4. How easy was it to correct mistakes?

very difficult — difficult — easy — very easy

|--------|-------|--------|-------|--------|-------|
1 2 3 4 5 6 7

5. How easy was it to use the included Rating Scales?

very difficult — difficult — easy — very easy

|--------|-------|--------|-------|--------|-------|
1 2 3 4 5 6 7

6. How easy was the software program to use in the presence of patients?

very difficult — difficult — easy — very easy

|--------|-------|--------|-------|--------|-------|
1 2 3 4 5 6 7

7. How easy was the software program to use with concurrent demands?

very difficult — difficult — easy — very easy

|--------|-------|--------|-------|--------|-------|
1 2 3 4 5 6 7

8. How easy was the software program to use with your daily workflow?

very difficult — difficult — easy — very easy

|--------|-------|--------|-------|--------|-------|
1 2 3 4 5 6 7

9. How easy was it to prescribe medications using the software program?

very difficult — difficult — easy — very easy

|--------|-------|--------|-------|--------|-------|
1 2 3 4 5 6 7

10. How easy was it to change the stage in the algorithm?

very difficult — difficult — easy — very easy

|--------|-------|--------|-------|--------|-------|
1 2 3 4 5 6 7

11. Is the terminology similar to that used in your work?

not at all similar — slightly similar — mostly similar — very similar

|--------|-------|--------|-------|--------|-------|
1 2 3 4 5 6 7

12. Was the speed of the screen loads acceptable?

not at all acceptable — slightly acceptable — mostly acceptable — very acceptable

|--------|-------|--------|-------|--------|-------|
1 2 3 4 5 6 7

Algorithm Evaluation Questionnaire: Software Application Version
Part II: Usefulness

Directions: We are interested in your opinion on the usefulness of this software program. Please circle the number that corresponds with your opinion on this software program.

1. Overall how useful was the software program?

not at all useful — slightly useful — mostly useful — very useful

|--------|-------|--------|-------|--------|-------|
1 2 3 4 5 6 7

2. How useful was the software program in your everyday work?

not at all useful — slightly useful — mostly useful — very useful

|--------|-------|--------|-------|--------|-------|
1 2 3 4 5 6 7

3. Did you find the treatment recommendations provided by the program useful?

not at all useful — slightly useful — mostly useful — very useful

|--------|-------|--------|-------|--------|-------|
1 2 3 4 5 6 7

4. To what extent has the use of the software program improved your productivity?

not at all improved — slightly improved — mostly improved — very much improved

|--------|-------|--------|-------|--------|-------|
1 2 3 4 5 6 7

5. How often did you use the "Override" function?

never — seldom — sometimes — often

|--------|-------|--------|-------|--------|-------|
1 2 3 4 5 6 7

6. How useful were the Warnings and Prompts?

not at all useful — slightly useful — mostly useful — very useful

|--------|-------|--------|-------|--------|-------|
1 2 3 4 5 6 7

7. How would you rate the program's ability to cover the tasks necessary to conduct a patient visit?

Does not cover — slightly covers — mostly covers — completely covers

|--------|-------|--------|-------|--------|-------|
1 2 3 4 5 6 7

8. How useful was the software program in the prescribing of medication?

not at all useful — slightly useful — mostly useful — very useful

|--------|-------|--------|-------|--------|-------|
1 2 3 4 5 6 7

9. How useful was the software program in following a patient's status over time?

not at all useful — slightly useful — mostly useful — very useful

|--------|-------|--------|-------|--------|-------|
1 2 3 4 5 6 7

10. Did the software improve the quality of patient care that you were able to provide?

not at all improved — slightly improved — mostly improved — very much improved

|--------|-------|--------|-------|--------|-------|
1 2 3 4 5 6 7

11. Is the software program flexible enough to use with the types of patients you see?

not at all flexible — slightly flexible — mostly flexible — very flexible

|--------|-------|--------|-------|--------|-------|
1 2 3 4 5 6 7

12. Is using this software program educational in regard to the treatment of patients?

not at all educational — slightly educational — mostly educational — very educational

|--------|-------|--------|-------|--------|-------|
1 2 3 4 5 6 7

13. Does the software ever suggest treatments that you might not have used otherwise?

never — seldom — sometimes — often

|--------|-------|--------|-------|--------|-------|
1 2 3 4 5 6 7

14. Does the software reduce the time required to determine treatment?

never — seldom — sometimes — often

|--------|-------|--------|-------|--------|-------|
1 2 3 4 5 6 7

15. Does the software draw your attention to aspects or problems with the patient that you might not have noticed otherwise?

never — seldom — sometimes — often

|--------|-------|--------|-------|--------|-------|
1 2 3 4 5 6 7

16. Does the software ever reduce the need for assistance from other clinical professionals?

never — seldom — sometimes — often

|--------|-------|--------|-------|--------|-------|
1 2 3 4 5 6 7

17. Do you think using the software is helpful in reducing the risk of error?

not at all helpful — slightly helpful — mostly helpful — very helpful

|--------|-------|--------|-------|--------|-------|
1 2 3 4 5 6 7

18. Is the software helpful in educating patients?

not at all helpful — slightly helpful — mostly helpful — very helpful

|--------|-------|--------|-------|--------|-------|
1 2 3 4 5 6 7
